# Structural determinants of ligand response specificity in the mast cell activating GPCR, MRGPRX2

**DOI:** 10.1016/j.jbc.2026.113132

**Published:** 2026-05-08

**Authors:** Abiodun Adefola R. Adeosun, Melina A. Agosto, Olivier Lichtarge, Theodore G. Wensel

**Affiliations:** 1 Department of Biochemistry and Molecular Pharmacology, Baylor College of Medicine, Houston, Texas, USA; 2 Department of Physiology and Biophysics, Halifax, Canada; 3 Retina and Optic Nerve Research Laboratory, Department of Ophthalmology and Visual Sciences, Dalhousie University, Halifax, Canada; 4 Department of Molecular and Human Genetics, Baylor College of Medicine, Houston, Texas, USA

**Keywords:** Evolutionary Action, G-protein coupled receptor, mast cell activation, MRGPRX2, single nucleotide polymorphism

## Abstract

The mast cell-specific G-protein-coupled receptor (GPCR) MRGPRX2 (Mas-Related G Protein-coupled Receptor X2) has roles in itch and pain, and it mediates clinically relevant allergy-like responses to a diverse assortment of drugs. The varied responses of individuals to MRGPRX2 agonists, leading to drug hypersensitivity reactions in some cases, suggests the presence of consequential variants in the population. However, genetic associations with drug responses are poorly understood. We used heterologously-expressed MRGPRX2 to investigate the effect of 18 naturally occurring non-synonymous single nucleotide polymorphisms on activation by representative compounds from several classes, including neuropeptides, opioid agonists, antibiotics, neuromuscular blocking agents, and polycationic aromatic compounds. The results demonstrate robust activation of wild-type MRGPRX2 by some, but not all, agonists of each class, and not by agonists of closely related MRGPRX1. Naturally occurring MRGPRX2 amino acid substitutions had a range of effects on the potency of different agonists, including loss of function and gain of function phenotypes. The MRGPRX family has diverged from other Class A GPCRs at sites canonically important for receptor activation. Deleterious mutations were found in canonical GPCR functional sites, including those where the MRGPRX family has divergent sequences, as well as in novel functional sites. Severe loss-of-function mutations were generally scored as likely to be deleterious by the Evolutionary Action algorithm while gain-of-function mutations had low to moderate scores. The results will inform future studies aimed at developing drugs targeting MRGPRX2 and uncovering disease-relevant mutations.

Mast cells are critical components of the immune system, playing essential roles in both innate and adaptive immunity (1, 2, 3, 4). Beyond their immunoprotective functions, mast cells have also been implicated in the pathophysiology of numerous diseases, including pseudo-allergic reactions to drugs (5, 6, 7, 8), chronic idiopathic urticaria (9), atopic dermatitis (10, 11), asthma (11), chronic itch (12), and chronic pain (13, 14). Mast cell activation is a receptor-mediated, pro-inflammatory response that occurs *via* the IgE receptor (15) or the Mas-related G-protein coupled receptor X2 (MRGPRX2) (16, 17, 18, 19). In response to activation, the critical function of mast cells is the secretion of cytoplasmic granules containing diverse inflammatory mediators.

IgE-independent mast cell activation has long been observed (20, 21), particularly in response to cationic substances such as neuropeptides (14, 22), antimicrobial peptides (23), and various drugs (5). Unlike IgE-mediated reactions, these responses did not require prior sensitization. A breakthrough in understanding this alternative pathway came with the discovery of the MRGPRX receptor family (13), and the subsequent identification of MRGPRX2 as a key receptor mediating IgE-independent mast cell activation (5, 19, 23). The role of MRGPRX2 in drug hypersensitivity reactions (DHRs) is of particular interest and clinical relevance, because DHRs can be severe and occur without prior sensitization (24).

MRGPRX2 is a Class A GPCR expressed on human mast cells, primarily those found in the skin and sensory neurons (9, 19). Activation of MRGPRX2 promotes IgE-independent mast cell activation (5, 19, 23), contributing to inflammatory responses. It is considered the ortholog of rodent protein, MRGPRB2, which knockout experiments have verified to be responsible for similar responses by mast cells in mice (5). While IgE-mediated degranulation mechanisms are well characterized (15), the signaling mechanisms underlying MRGPRX2-mediated degranulation remain poorly understood. Increase in cytoplasmic calcium is a key factor promoting mast cell degranulation (25, 26), and MRGPRX2 has been shown to couple to both G_q_ and G_i_, leading to calcium mobilization and degranulation (14, 27, 28). Activation of MRGPRX2 has been studied primarily in the context of exogenous compounds associated with mast cell-mediated hypersensitivity (5, 16). MRGPRX2 has a broad ligand selectivity, including neuropeptides, opioid agonists, antibiotics, neuromuscular blocking agents, and polycationic aromatic compounds and responds to many drugs only at micromolar concentrations and above (24, 29).

DHRs vary widely among individuals, and to date, no clear genetic associations have been established. While many naturally occurring variants of MRGPRX2 have been identified through genomic sequencing, there is little data linking these variants to individual susceptibility to mast cell hypersensitivity, with a few recent exceptions (27, 30, 31, 32). In this study, we used Evolutionary Action (EA) (33, 34) and previously reported structures of MRGPRX2 (28, 35), as well as canonical GPCR functional motifs, to identify single-nucleotide polymorphisms (SNPs) of interest. We characterized the functional impacts of variants using a cell-based calcium mobilization assay and a panel of diverse ligands. We report deleterious naturally occurring MRGPRX2 amino acid substitutions at canonical GPCR functional sites, including in motifs that are divergent in the MRGPRX family, while substitutions at other positions had varying effects depending on the ligand.

## Results

### MRGPRX2 can be activated by structurally diverse compounds with a wide range of potencies

For this study, we selected compounds that represent a variety of molecules, including antibiotics, polybasic compounds, opioids, and endogenous peptides that have been reported to be mast cell activators, MRGPRX2 agonists, or MRGPRX1 agonists (29) (Fig. S1). We also included some clinically important drugs previously reported to have low potencies or to be non-activators (36), because they are administered through injections or are applied topically (*e.g.* polymyxins, ciprofloxacin), such that MRGPRX2-expressing mast cells in the skin are exposed to high local concentrations. Surprisingly, we found that some of them, as detailed below, are robust agonists of MRGPRX2.

To establish baseline comparisons among ligands using a single quantitative assay, we used a fluorescence-based Ca^2+^ mobilization assay in transfected HEK293 cells, which reflects activation of endogenous G_αq_. The advantage of this system is that it allows us to test multiple ligands in a cell line that lacks endogenous MRGPRX2 expression, while retaining the essential components required for MRGPRX2-mediated signaling (37). Importantly, the HEK293 cell line also lacks other known receptors for the tested ligands, ensuring that responses are specific to MRGPRX2. This specificity was confirmed by testing responses to all compounds in cells transfected with empty vector (EV) control (Figs. S2 and S3).

**Figure 1:**
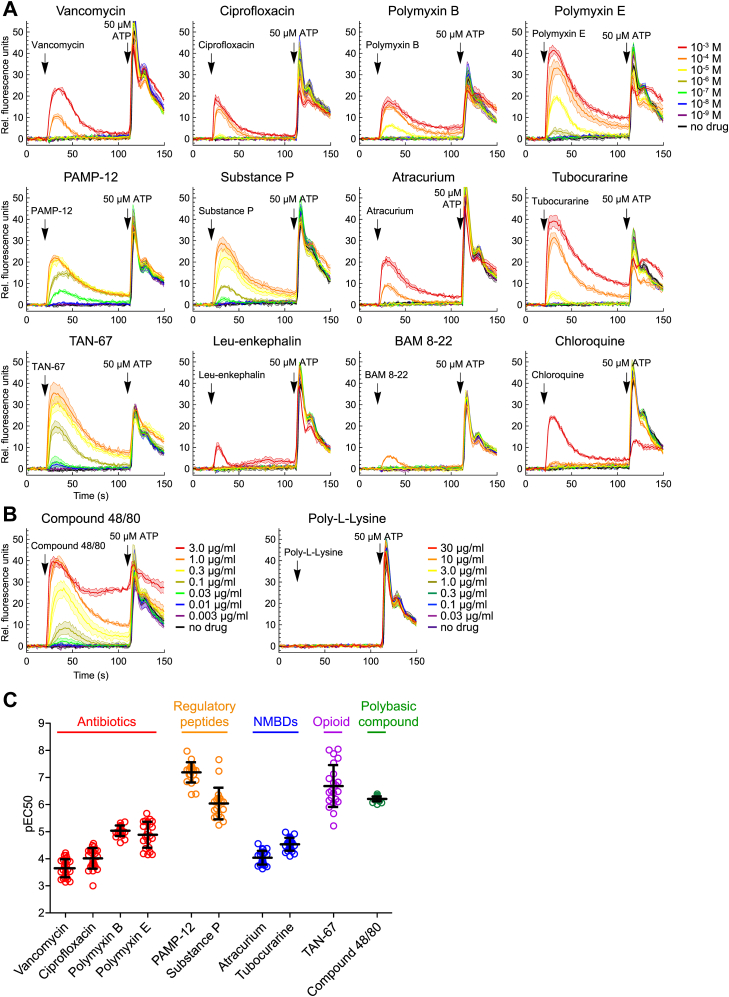
**MRGPRX2-mediated Ca^2+^ mobilization in transfected HEK293 cells.***A* and *B*, representative Ca^2+^ mobilization traces showing responses of WT MRGPRX2 to different ligands as indicated. Arrows indicate injection of agonist at 20 s, followed by injection of 50 μM ATP at 110 s as a positive control. Shaded regions indicate s.d. of technical replicates. Negative control traces from cells transfected with empty vector are shown in Figure S2. *C*, pEC50 values from dose response curves derived from peak amplitudes of traces as shown in *A*. Example dose response curves are shown in Figure S3. Each point represents three technical replicates in an independent experiment (n = 20–25). Bars show overall mean ± s.d. For comparison, EC50 values for compound 48/80 were converted to molar quantities using the molecular weight of a trimer.

HEK293 cells were transiently transfected with plasmids encoding WT MRGPRX2 or EV. Receptor activation was then monitored in real time by increased fluorescence of the cytoplasmic Ca^2+^-sensitive dye, fluo-4, upon ligand addition (Figs. 1, *A* and *B*, S3). As a positive control, subsequent addition of ATP induced Ca^2+^ mobilization across all conditions, indicating a functional G_αq_-mediated calcium response *via* the endogenous purinergic receptor (Figs. 1 and S2). EV controls confirmed the absence of endogenous responses to any of the ligands used in HEK293 cells, except for large rapid responses to the highest concentration of TAN-67 (1 mM, therefore excluded from all analyses), and delayed responses to the highest concentrations of polymyxin B (1 mM) and Compound 48/80 (3 μg/ml) (Fig. S2) (see discussion below for compound 48/80). Potencies were derived from sigmoidal dose–response curves fit to peak activation values (Fig. S3), and pEC50 values are shown in Figure 1*C* and Table 1. In cases for which dose–response curves did not approach saturation sufficiently for robust determination of EC50 values, the best-fit values are listed as upper limits.

**Table 1:** Agonist potency values for WT MRGPRX2 in Ca^2+^ mobilization assays

Agonist	*n*^a^	pEC50 (–Log_10_[M])^b^	EC50 (μM)
Vancomycin	25	3.65 ± 0.33^c^	230^c^
Ciprofloxacin	24	4.01 ± 0.38	97
Polymyxin B	21	5.03 ± 0.19	9.3
Polymyxin E	22	4.88 ± 0.48	13
PAMP-12	21	7.19 ± 0.37	0.065
Substance P	20	6.04 ± 0.58	0.91
BAM 8–22	21	< ∼4^c^	> ∼100^c^
Chloroquine	21	< ∼3^c^	> ∼1000^c^
Atracurium	24	4.04 ± 0.25^c^	92^c^
Tubocurarine	23	4.53 ± 0.24	29
TAN-67	22	6.68 ± 0.77	0.21
Leu-Enkephalin	21	< ∼3^c^	> ∼1000^c^
Compound 48/80	21	∼6.2–6.5 (0.55 ± 0.09, –Log_10_[μg/ml])	∼0.31–0.62 (0.28 μg/ml)
Poly-l-Lysine	21	NR	NR

a Number of independent experiments.

b mean ± SD; NR, no response.

c indicates pEC50 values are upper limits (EC50 values are lower limits), because dose response curves did not saturate.

#### Responses to antibiotic peptides

Antibiotics, including cationic peptides such as vancomycin and polymyxins, along with fluoroquinolones, such as ciprofloxacin, are known to activate MRGPRX2 and have been implicated in non-IgE-mediated DHRs (38). Polymyxin B and polymyxin E, closely related polycationic peptides that differ in structure only by a single amino acid in the heptapeptide ring (Fig. S1), activated WT MRGPRX2 with similar potencies—pEC50 values were 5.03 ± 0.19 and 4.88 ± 0.48, respectively. The response to polymyxin B was abolished in G_αq/11_ knockout HEK cells (39) transfected with MRGPRX2, and was rescued by co-transfecting G_αq_ with MRGPRX2 (Fig. S4). These results demonstrate that G_αq/11_ is required for MRGPRX2-mediated Ca^2+^ mobilization in response to polymyxin B.

The cyclic glycopeptide antibiotic vancomycin, well known for severe mast cell mediated inflammatory responses linked to MRGPRX2 (8, 40, 41), activated MRGPRX2 with a potency that is at least an order of magnitude lower than the polymyxins (upper limit of pEC50 = 3.65 ± 0.33); accurate pEC50 values could not be calculated because the dose response curve showed no signs of saturation at a concentration of 1 mM (Fig. S3).

#### Responses to neuropeptides and opioid agonists

Substance P is an endogenous neuropeptide involved in pain, inflammation, and neuroimmune signaling and a well-established potent agonist of MRGPRX2 (22, 42). In our assay, it exhibited a pEC50 of 6.04 ± 0.58. PAMP-12, another endogenous peptide, is derived from the adrenomedullin precursor, proadrenomedullin, and is known to induce MRGPRX2-mediated mast cell degranulation (43). Of all the agonists tested, PAMP-12 was the most potent activator of MRGPRX2-mediated Ca^2+^ release, with a pEC50 of 7.19 ± 0.37.

The opioid peptide Leu-enkephalin, a potent agonist at the δ and μ opioid receptors, elicited MRGPRX2 responses only at the highest concentration (1 mM) (Figs. 1 and S3). The peptide BAM 8-22 (bovine adrenal medulla peptide 8–22), a potent activator of MRGPRX1 (44, 45, 46), resulted in only weak MRGPRX2 responses at 0.1 mM, similar to vancomycin responses. We were not able to test BAM 8-22 at 1 mM. However, in line with the suggestion that MRGPRX2 is an atypical opioid receptor, and consistent with prior reports (36), the small molecule δ-opioid agonist, TAN-67 (SB-205607), was among the most potent agonists tested (pEC50 is 6.68 ± 0.77).

#### Responses to polycationic compounds

The classic mast-cell activator, compound 48/80, an oligomeric amino phenolic compound, consistently activated MRGPRX2 with high potency, with pEC50 (−log_10_[μg/ml]) of 0.55 ± 0.09, corresponding to pEC50 (−log_10_ [M]) of ∼6.2 to 6.5). The EC50 value is consistent with previous reports of MRGPRX2-mediated responses to compound 48/80 in heterologous cells (5, 41). At the highest concentration tested (3 μg/ml ≈ 3.3–6.5 μM), there was a delayed Ca^2+^ response in EV control assays (Fig. S2). However, a reliable dose response curve could be recovered by restricting the domain for extraction of peak amplitudes to the first 20 s after ligand addition (Figs. 1*B* and S3). As multiple polycationic compounds have been reported to activate MRGPRX2, and because of concern that use of poly-llysine to coat microtiter plate wells might give rise to background levels of activation or desensitization, we tested poly-l-lysine in the functional assay. Poly-l-lysine did not give rise to detectable activation at any concentration tested, demonstrating that concentrated positive charge is not sufficient for MRGPRX2 activation.

Chloroquine was reported to activate MRGPRX1 in HEK cells with low potency of ∼0.3 mM (47). Chloroquine contains aromatic rings and three basic nitrogen atoms, with considerable flexibility in its aliphatic region, and thus might be expected to assume conformations conferring at least some affinity for the shallow MRGPRX2 ligand binding site (see Discussion). The activation of MRGPRX2 by chloroquine appears to be similar to its activation of MRGPRX1, with responses to 1 mM, but not 0.1 mM. The fluoroquinolone antibiotic ciprofloxacin also includes aromatic rings in close proximity to a basic group and displays only modest agonism for MRGPRX2 with a pEC50 of 4.01 ± 0.38.

#### Responses to neuromuscular blocking drugs

Hypersensitivity reactions to neuromuscular blocking drugs remain a significant challenge for surgical procedures relying on such drugs (48, 49, 50). The responses of MRGPRX2 to atracurium besylate (pEC50 ≤ 4.04 ± 0.25) and tubocurarine (pEC50 is 4.53 ± 0.24) at concentrations comparable to those encountered at administration sites highlight its important role in mediating these reactions. The only commonalities in the structures of these two drugs are the presence of two positively charged nitrogen atoms and multiple aromatic ether groups. They differ strikingly in their conformational degrees of freedom available *via* rotations about single bonds: atracurium contains many such degrees of freedom, whereas tubocurarine is a highly constrained ring structure. This distinction does not appear to have much impact on their potencies, in contrast to what might be expected for the increased entropic cost of binding atracurium in a fixed pose, as their EC50 values differ by only about a factor of 3.

### Naturally occurring SNPs within the MRGPRX2 receptor

**Figure 2:**
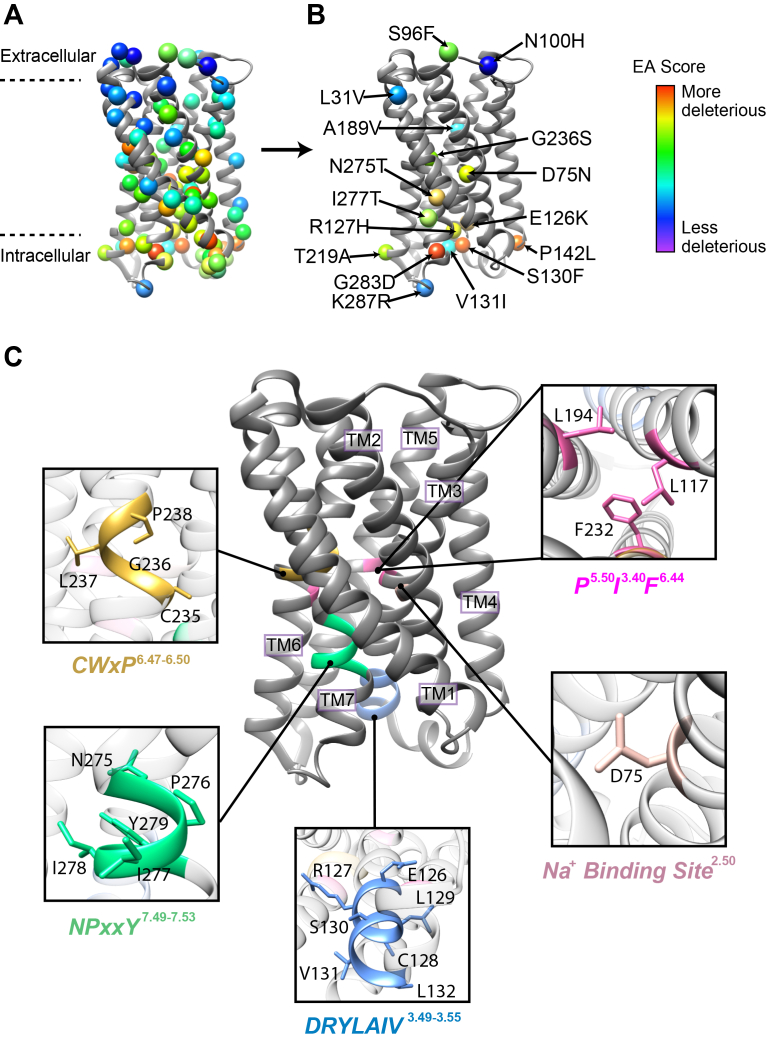
**MRGPRX2 SNPs and functional motifs.***A*, location of missense variants in the structure of MRGPRX2 (PDB 7VV4 (35)). P322 is not visible in the structure. *B*, positions of the 18 variants selected for testing. In (*A*) and (*B*), spheres are colored according to EA score; when multiple variants occur at the same residue, EA score of the first mutation in alphabetical order is shown. *C*, structural features and divergence of conserved Class *A* GPCR motifs in MRGPRX2. Magnified panels display the specific amino acid residues present at these motifs in MRGPRX2. Motif names correspond to the standard one-letter amino acid abbreviations, with "x" indicating any amino acid at that position.

To conduct a search for consequential SNPs in MRGPRX2, we started with the well-established Evolutionary Trace (ET) method (51, 52), which identifies important sequence positions at which divergence of an evolutionary tree is associated with fixation of residue identity, and the Evolutionary Action (EA) algorithm (34), which integrates ET with the likelihood of specific amino acid substitutions based on substitution frequency. Together, these factors yield a quantitative estimate of the functional impact of variants. EA scores range from 0 (predicted to be benign) to 100 (predicted to be highly deleterious). The ET scores of all MRGPRX2 residues are shown in Figure S5. We identified 197 naturally occurring amino acid substitutions caused by SNPs using the Genome Aggregation Database (GnomAD) (53). EA scores of the missense mutations, which are located throughout the MRGPRX2 structure (28), are shown in Figure 2*A* and listed in Table S1. Missense mutations chosen for further study are shown in Figure 2*B* and Table S2, and *italicized* in this section.

When choosing mutations, we also considered that canonical Class A GPCR motifs (54) may contribute to ligand responses in MRGPRX2. Comparisons between MRGPRX2 and other Class A GPCRs revealed that several of these motifs are either absent or altered in MRGPRX2 (Fig. 2*C*). The CWxP motif appears in MRGPRX2 as residues (updated Ballesteros-Weinstein numbering system (35, 55, 56)) C235^6.47^, G236^6.48^, L237^6.49^, P238^6.50^, with the typical W^6.48^ replaced by G236 in MRGPRX receptors. All four of the residues in this motif were identified as important by ET analysis (Fig. S5). Three naturally occurring mutations have been reported in this motif: one at G^6.48^ (*G236S*) and two at P^6.50^ (P238A and P238S). Interestingly, the toggle switch residue (W^6.48^), which is highly conserved in class A GPCRs (GPCRdb (56)), is typically substituted by either glycine or serine in human MRGPRs. It is noteworthy that the naturally occurring SNP *G236S*, despite serine being the WT residue in other MRGPR family members, still has a moderately high EA score of 63. The two SNPs at P^6.50^ are significant given that residue is nearly universally conserved (∼99%) across Class A GPCRs (GPCRdb (56)), and nearly every human MRGPR family member retains a proline at this position.

The conserved DRY motif, which plays a critical role in GPCR activation and G protein coupling (57, 58), appears in MRGPRX2 as E126^3.49^, R127^3.50^, and C128^3.51^. Interestingly, this noncanonical sequence is conserved in the MRGPRX family (including human proteins MRGPRF, MRGPRG, MRGPRX1, MRGPRX2, MRGPRX3, and MRGPRX4; replaced by QRC in MRGPRD and by EQC in MRGPRE; GPCRdb (56)). Many class A GPCRs contain an extended version of this motif, referred to as the DRYLAIV motif, which corresponds to E126, R127, C128, L129, S130, V131, and L132 in MRGPRX2. Within this motif, we identified eight SNPs. At E^3.49^, two SNPs were observed: *E126D*, with a moderate EA score of 57, and *E126K*, which had a high EA score of 82, suggesting potential functional significance. At R^3.50^, a highly conserved residue critical for G protein interaction, two substitutions were found: R127C and *R127H*, both of which were associated with high EA scores (88 and 73, respectively). At S^3.53^, a single variant, *S130F*, had an EA score of 92, again suggesting high impact. Lastly, V^3.54^ presented three naturally occurring variants: V131D (EA 91), *V131I* (EA 27), and V131L (EA 50).

The NPxxY motif is a highly conserved feature of class A GPCRs and plays a pivotal role in facilitating the conformational rearrangements associated with receptor activation (59, 60). In the MRGPRX family, this motif is represented by the sequence N^7.49^, P^7.50^, I^7.51^, I^7.52^ and Y^7.53^, corresponding to N275, P276, I277, I278 and Y279 in MRGPRX2. Within this motif, only two naturally occurring missense mutations have been reported: *N275T* and *I277T*, with EA scores of 81 and 63, respectively. Despite being a relatively conservative substitution, the *N275T* mutation still yields a high EA score, consistent with the high ET ranking (ET score: 95), underscoring its functional importance in receptor signaling. In contrast, the *I277T* mutation produces a more moderate EA score, reflecting both the less conserved nature of I^7.51^ and the more subtle impact of this substitution. These findings support the functional relevance of the NPxxY motif in MRGPRX2 and suggest that even conservative mutations within this region can significantly alter receptor behavior when they occur at highly constrained positions.

In class A GPCRs, the PIF motif (P^5.50^, I^3.40^, F^6.44^) forms a structurally conserved triad spanning helices 3, 5, and 6 (61). This motif plays a critical role in stabilizing the inactive conformation and undergoes rearrangement during receptor activation (62, 63). However, in MRGPRX2, this canonical PIF motif is notably absent. Instead, the corresponding positions are occupied by L194^5.50^, L117^3.40^, and F232^6.44^. A similar substitution is observed across other MRGPRX family members, where the motif appears as LLF rather than PIF. Despite the divergence from the canonical motif, all three residues (L117, L194, and F232) were assigned high ET scores, indicating they are evolutionarily conserved and likely functionally important within the MRGPRX lineage. This suggests that while the specific amino acid identities differ from typical Class A GPCRs, the structural or dynamic role of this region may be functionally conserved through alternative residues. No naturally occurring SNPs have been reported at these positions.

Sodium-binding pockets are a key structural feature associated with GPCR stabilization, typically formed by a set of highly conserved residues, including D^2.50^ (D75), S^3.39^ (G116), N^7.45^ (N271), S^7.46^ (S272), and Y^7.53^ (Y279) (64, 65). All five residues are highly conserved across the MRGPRX family. Of the five sodium pocket residues, only D^2.50^ had naturally occurring SNPs: D75E (EA 72) and *D75N* (EA 71). MRGPRX2 has been referred to as an atypical opioid receptor, prompting comparison to conserved structural features found in canonical opioid receptors. As indicated above, MRGPRX2 can be activated potently by TAN-67, a highly selective δ-opioid receptor agonist. In the δ-opioid receptor, N^3.35^, which forms a bridge between N^7.45^ and D^2.50^, has been shown to participate in Na^+^ coordination (64, 65, 66). While most class A GPCRs have a hydrophobic residue at this position, N^3.35^ is conserved among opioid receptors (66, 67). In contrast, MRGPRX2 possesses an alanine at the same position (A112^3.35^). Although no naturally occurring SNPs were reported at this position, experimental studies have demonstrated that substituting N^3.35^ in δ-opioid receptor with hydrophobic residues valine or alanine either reduced or abolished sodium-mediated allosteric effects on ligand binding (66). EA analysis was used to estimate the potential impact of substitutions to asparagine (A112N) and valine (A112V) in MRGPRX2; both variants yielded relatively low EA scores (22 and 19, respectively).

Several SNPs outside of conserved motifs were also included to ensure a wide range of EA scores. A naturally occurring SNP at L31^1.33^ (*L31V*) results in an EA score of 15. L31^1.33^ has a low ET score, suggesting a relatively low functional impact. This residue is conserved across the MRGPRX subfamily but not in the broader MRGPR family. At S96^2.71^ the *S96F* substitution was identified, with a moderate EA score of 52 and a low ET score. N100 (no corresponding Ballesteros-Weinstein number) has a low ET score, and three SNPs at this position all have low EA scores: N100D (EA 0.29), *N100H* (EA 0.53), and N100I (EA 17). At position P142^4.38^, a high-impact SNP (*P142L*) was identified, with an EA score of 90. This residue has a high ET score and is conserved across all human MRGPRs except MRGPRX4. At A189^5.45^, a single SNP (*A189V*, EA 24) was identified. T219^6.31^ has a SNP (*T219A*) with a moderate EA score of 65. G283^7.57^ has a potentially high-impact SNP (*G283D*) with a high EA score of 94. This residue is conserved in the MRGPRX subfamily, though no Ballesteros-Weinstein number exists in the broader MRGPR family. The SNPs *K287R* (EA 15) and *P322A* (EA 17) both had low ET scores in addition to their low EA scores.

In summary, EA scores and positions within known canonical motifs were considered when selecting SNPs for testing. We included both moderate and high-scoring variants to assess a range of predicted functional impacts, along with several low-scoring variants to serve as internal controls. A total of 18 SNPs were chosen for further analysis (Table S2). Their positions in the MRGPRX2 structure are shown in Figure 2*B*.

### MRGPRX2 missense mutations confer loss-of-function and gain-of-function phenotypes

We first tested the ability of MRGPRX2 mutants to be synthesized and trafficked to the cell surface in HEK293 cells. Transfected cells were labeled with a FLAG antibody in nonpermeabilizing or permeabilizing conditions, to detect surface and total expression, respectively (Figs. S6 and S7). Surface expression of mutants varied widely, with most between ∼20 to 80% of WT levels. Exceptions were N275T, whose surface expression was comparable to or greater than WT, and E126K, P142L, I277T, and G283D, which had severely reduced surface expression. To assess the impact of the SNPs on G protein activation, all the mutants were tested in the Ca^2+^ mobilization assay using the entire panel of ligands (Fig. S1). Representative traces and dose response curves are shown in Figures S8, 3 and 4. Maximum responses to the highest drug concentration are shown in Figure S9. However, because response amplitudes, or efficacy, depend on the amount of receptor at the cell surface, we focused instead on measuring the potency of different ligands for each mutant (Figs. 5 and 6, Table S3) to minimize sensitivity to surface expression levels.

**Figure 3:**
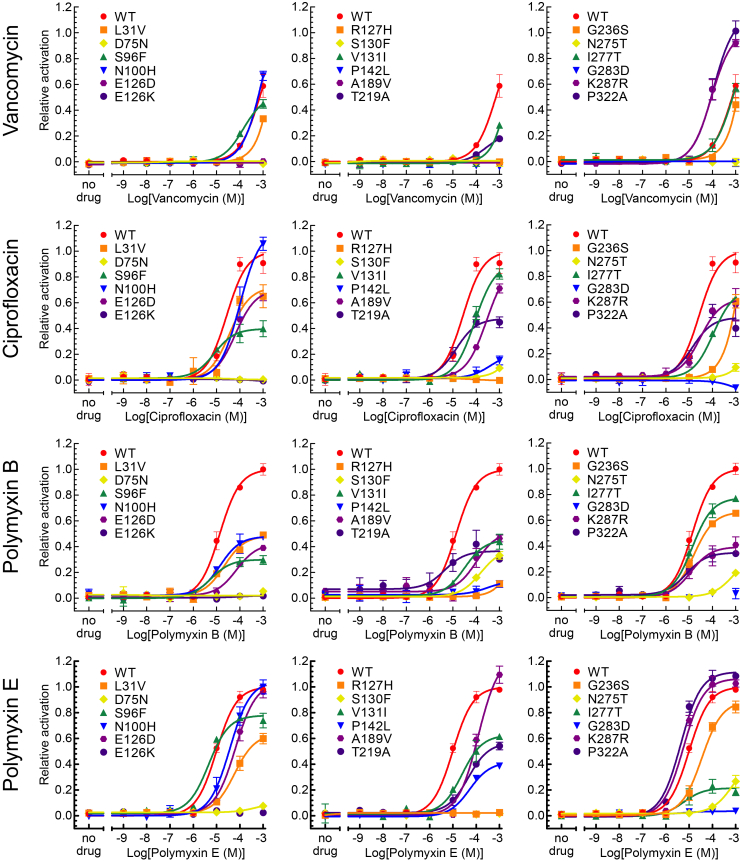
**Activation of MRGPRX2 variants in response to antibiotics.** Representative dose response curves generated from Ca^2+^ mobilization assays. Example traces are shown in Figure S8, *A–D*. Points represent the mean ± SD of three technical replicates, and curves are normalized to the maximal response observed for WT on the same plate.

**Figure 4:**
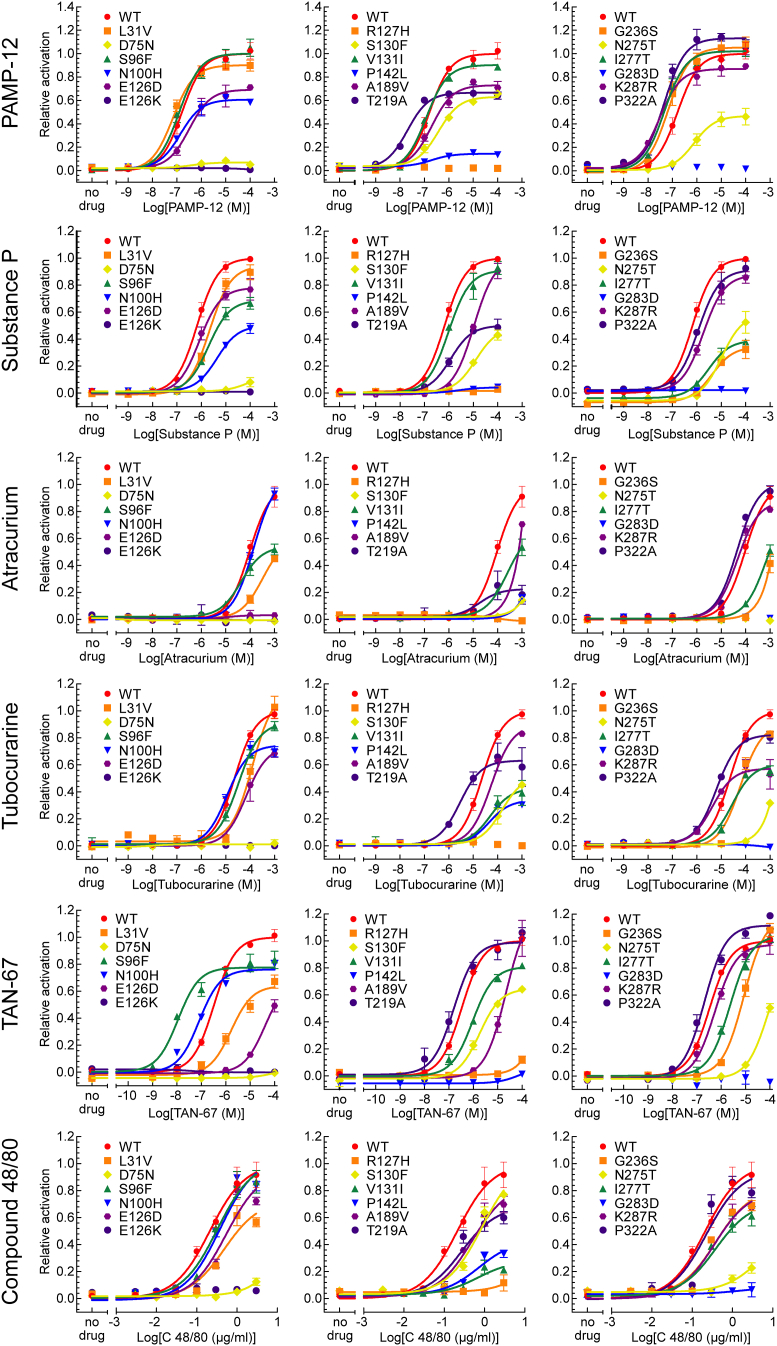
**Activation of MRGPRX2 variants in response to endogenous peptides, compound 48/80, neuromuscular blocking drugs, and TAN-67.** Representative dose response curves generated from Ca^2+^ mobilization assays. Example traces are shown in Figure S8, *E–M*. Points represent the mean ± SD of three technical replicates, and curves are normalized to the maximal response observed for WT on the same plate.

**Figure 5:**
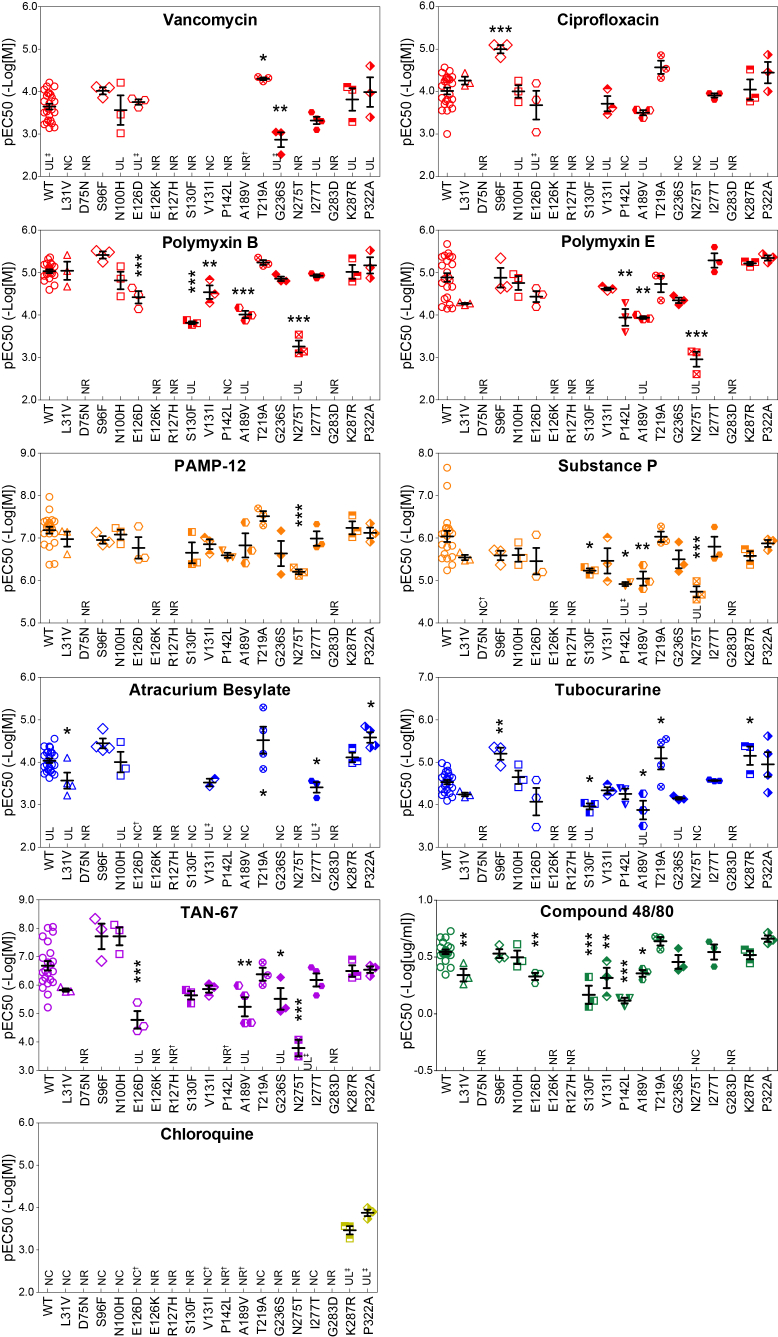
**pEC50 values for activation of MRGPRX2 variants by different agonists.** Values were derived from dose-response curves generated by measuring intracellular calcium following stimulation with agonist. Each point represents an independent experiment, and bars shown overall mean ± SEM. Conditions that had no response at any drug concentration (NR), for which low potencies prevented fitting of dose response curves (NC), or whose pEC50s represent upper limits (UL), are indicated. ^†^, The majority response is shown, but replicates varied between NR and NC. ^‡^, One or more additional replicates were NC. For TAN-67, artifacts were observed with 1 mM, so mutants responding only at 1 mM were scored as NR. Mutants with pEC50 values were compared to WT by one-way ANOVA and Dunnett’s post-test: ∗*p* < 0.05; ∗∗*p* < 0.01; ∗∗∗*p* < 0.001. Data for WT are the same as shown in Figure 1*C*.

**Figure 6:**
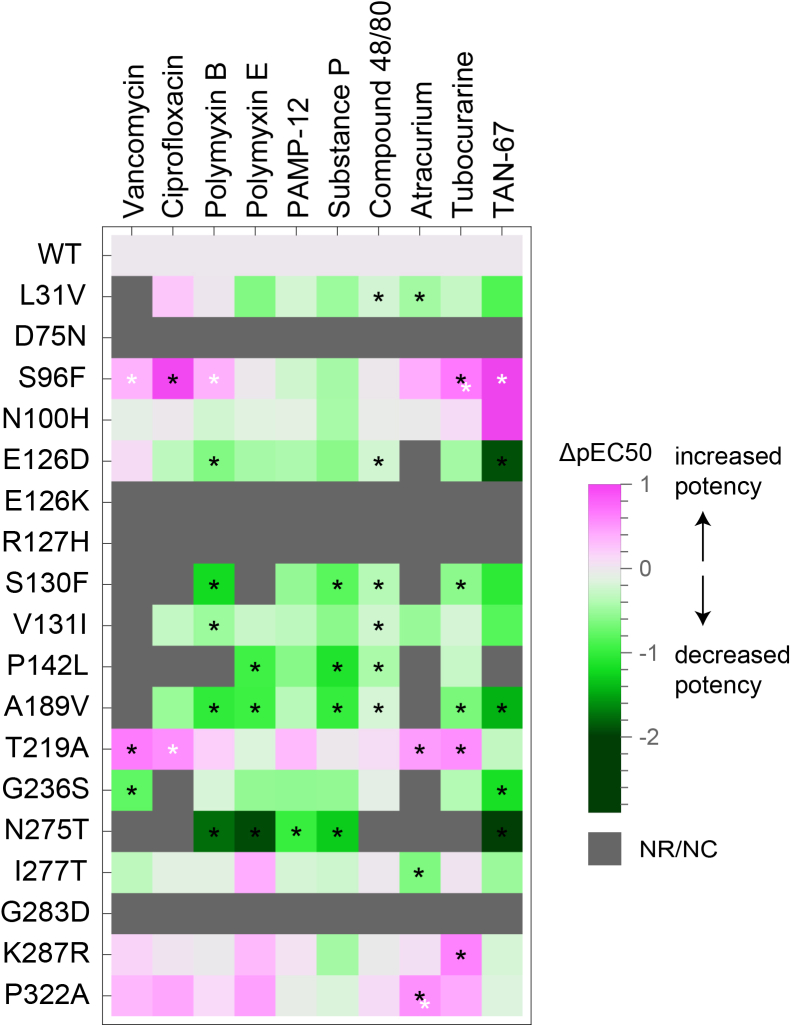
**Summary of changes in potency of different agonists at MRGPRX2 variants.** Squares are colored according to ΔpEC50 values (pEC50_mut_ - pEC50_WT_). Conditions which resulted in no measurable pEC50 are colored *grey*. *Black asterisks* indicate mutants that were significantly different from WT in one-way ANOVA (see Fig. 5). *White asterisks* indicate ligands for which gain of function variants were significantly different from paired WT values from the same plate (see Fig. 8).

Most variants responded to most agonists with EC50 values similar to those measured for WT (Figs. 5 and 6). The E126K and R127H variants, corresponding to E126^3.49^ and R127^3.50^ in the DRY motif (ERC in MRGPRX), were not responsive to any ligand. In the case of E126K, this lack of response may be explained by a lack of receptors at the cell surface. However, the surface expression of R127H was ∼50% of WT levels. S130F, also in the DRYLAIV motif, was generally deleterious, abolishing activity or reducing potency for most ligands. These results likely reflect the functional importance of the noncanonical DRY motif in MRGPRX2, and are consistent with the high EA scores of these substitutions. Mutation of the Na^+^ coordinating residue D^2.50^ (D75N) and G283D, which both had low surface expression, also abolished all activity. However, I277T, whose surface expression level was as low or lower than that of G283D (Fig. S7), responded to every ligand to which WT responds, suggesting that even low amounts of surface receptors are sufficient to produce measurable responses (Fig. 5). P142L, like E126K, had barely detectable surface expression. However, potencies could be measured for five ligands, three of which were significantly reduced. A189V, which had robust surface expression, had generally similar responses as P142L and S130F – no response to vancomycin, responses to only the highest concentration of atracurium, and reduced potency for most other ligands. However, S130F and P142L differed markedly in their response to the polymyxins (see below).

**Figure 7:**
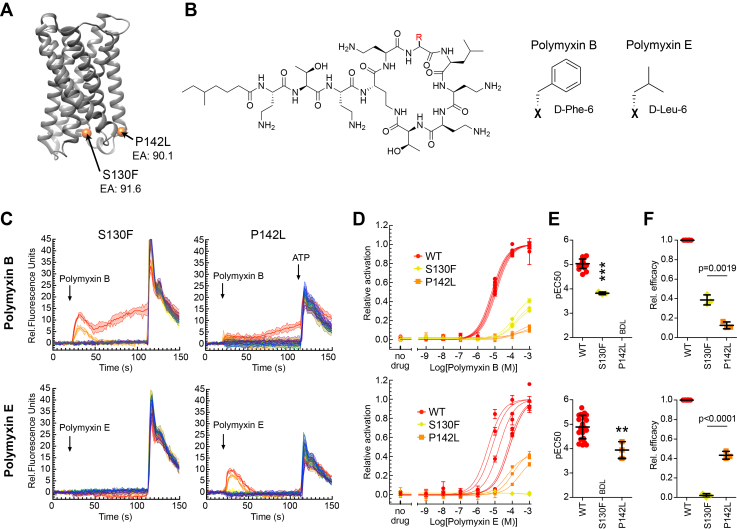
**MRGPRX2 variants altering specificity for polymyxins.***A*, structure of MRGPRX2 (PDB:7VV4 (35)) highlighting the positions of P142L and S130F, shown as *spheres* color-coded according to EA score as in Figure 2. *B*, chemical structure of polymyxin, showing the variable R group position where either a phenylalanine (polymyxin B) or a leucine (polymyxin E) is incorporated. *C*, Representative calcium traces from cells transfected with P142L or S130F, and stimulation with polymyxin *B* (*top*) or polymyxin E (*bottom*). *D*, Dose–response curves from traces as shown in (*C*), along with WT curves from the same plate. Points represent the mean ± SEM of three technical replicates and curves are normalized to the maximal response observed for WT on the same plate. *E*, pEC50 values, reproduced from Figure 5 to highlight P142L and S130F. *F*, relative efficacy values, derived from the plateaus of the dose response curve fits shown in (*D*). For the efficacy of polymyxin E for S130F, the difference between the maximum response and the no-drug condition was used. P142L and S130F values were compared with 2-tailed unpaired t tests. In (*E*) and (*F*), each point represents an independent experiment, and bars show mean ± SD.

Both polymyxin peptides activated WT MRGPRX2 with similar potencies. Several variants with moderate EA scores, including S96F, T219A, G236S and I277T, exhibited pEC50 values similar to those for WT, indicating preserved sensitivity to polymyxins. Similarly, variants predicted to be benign, including L31V, L100H, K287R, and P322A, also retained WT-like responses. A189V (EA score: 24) and N275T (EA score: 81) exhibited significantly reduced responses to both polymyxins, with pEC50 values approximately 10-fold and 100-fold lower than WT, respectively (Fig. 5). P142L^4.38^ (EA 90) and S130F^3.53^ (EA 92) revealed significant differences in the interactions of these two polymyxins with MRGPRX2 (Fig. 7). Polymyxin E activated P142L with a ∼10-fold reduced potency and ∼40% efficacy, while polymyxin B produced no measurable response. Conversely, Polymyxin B activated S130F with a similarly ∼10-fold reduced potency and ∼40% efficacy, while response to Polymyxin E was undetectable. These distinct activation profiles are striking given that Polymyxin B and E differ by only a single residue, suggesting subtle but critical ligand-receptor interaction differences.

Unsurprisingly, N275T (N^7.49^ in the NPxxY motif) was highly deleterious, either abolishing responses or significantly reducing potency for every ligand. In contrast, I277T (I^7.51^, also in the NPxxY motif), had little effect, with a significant change only for atracurium. G236S, located at the position of W^6.48^ in the CWxP motif, reduced the potency of ciprofloxacin and atracurium to non-measurable levels, and significantly reduced the potency of vancomycin and TAN-67 but had no effect on the potency of polymyxin B or compound 48/80.

**Figure 8:**
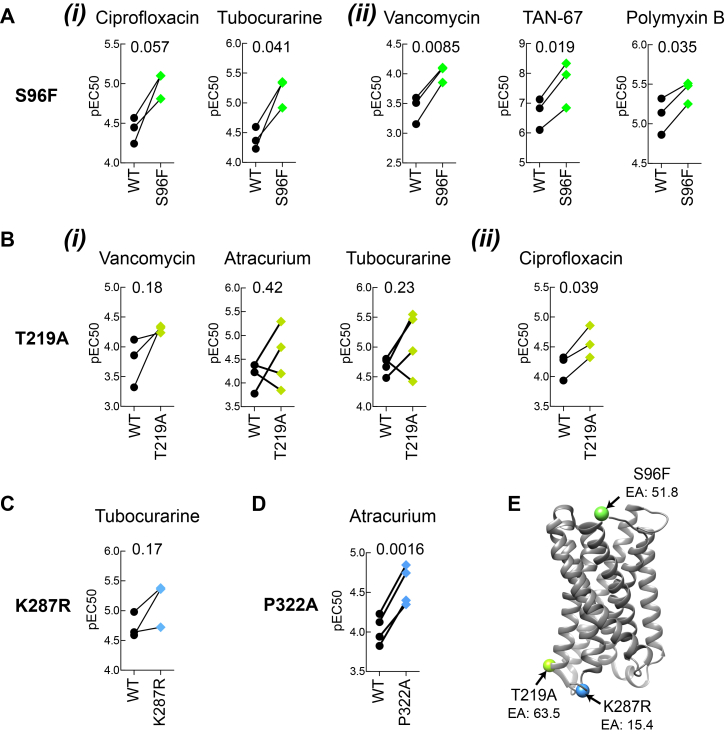
**Gain-of-function phenotypes of MRGPRX2 variants.***A–D*, comparison of WT and mutant pEC50 values from the same plate. Variants were compared to WT using 2-tailed paired t tests, and *p*-values are indicated above each plot. Points were calculated from three technical replicates; dose response curves are shown in Figure S10. *E*, structure of MRGPRX2 (PDB:7VV4 (35)), highlighting the positions of S96F, T219A, and K287R, shown as spheres color-coded according to EA score as in Figure 2. P322A is not visible in the structure.

Some gain-of-function phenotypes in the form of increased agonist potency were also observed (Figs. 6 and 8). The T219A variant exhibited a general trend towards increased potency, with significant increases for vancomycin (ΔpEC50 = 0.65), atracurium besylate (ΔpEC50 0.48), and tubocurarine (ΔpEC50 0.56). Similarly, S96F, K287R, and P322A conferred either little change or increased potency, with a statistically significant increase in potency for at least one agonist (Fig. 6). S96F exhibited an order of magnitude increase in potency of ciprofloxacin and a smaller increase for tubocurarine (ΔpEC50 0.99 and 0.67, respectively). The mean TAN-67 potency at S96F was also an order of magnitude higher than for WT, but the difference did not reach statistical significance in the ANOVA (Fig. 5) (ΔpEC50 1.0). K287R increased the potency of tubocurarine (ΔpEC50 0.62), while P322A increased the potency of atracurium (ΔpEC50 0.55). Interestingly, significant increases in potency were primarily observed for antibiotics vancomycin and ciprofloxacin, and for the neuromuscular blocking drugs, atracurium and tubocurarine. P322A and K287R also appeared to confer a gain of function for responses to BAM 8-22 and chloroquine. Although no BAM 8-22 pEC50s could be calculated for either WT or mutants, the P322A and K287R variants generated reproducibly larger responses to 0.1 mM (Figs. S9 and S10). For chloroquine, whereas pEC50 could not be calculated for WT, the P322A and K287R variants yielded dose response curves and upper limit pEC50s (Fig. 5).

To further assess the gain-of-function phenotypes, we examined the replicate dose response curves, comparing mutant and WT MRGPRX2 from the same plate (Figs. 8 and S10). Based on this analysis, the potency of ciprofloxacin and tubocurarine was possibly increased at the S96F variant (*p* values 0.057 and 0.041, respectively) (Fig. 8*A*i). In addition, we identified potential increased potency for three other ligands – vancomycin (*p* = 0.0085), TAN-67 (*p* = 0.019), and polymyxin B (*p* = 0.035) (Fig. 8*A*ii). The T219A variant did not exhibit significantly increased potency for the three ligands identified by ANOVA (Fig. 8*B*i). However, a potential increase in potency was identified for ciprofloxacin (Fig. 8*B*ii). The increased potency of atracurium for P322A (*p* = 0.0016) was also evident in this analysis (Fig. 8*D*).

## Discussion

Important open questions about MRGPRX2 function include (1) how activation is mediated by such structurally diverse agonists, and (2) how MRGPRX2 responses to agonists are dramatically different in some individuals. In this study, we characterized the response of 18 naturally occurring missense variants to a panel of ligands. We identified variants that resulted in complete loss of function across all ligands, revealing amino acids unequivocally required for receptor function. We also identified variants that resulted in altered potency for only some ligands, suggesting distinct allosteric activation pathways in the MRGPRX2 protein. Several gain-of-function variants, which could possibly contribute to DHRs, were also identified.

Despite high sequence similarity within the MRGPR subfamily, distinct ligand specificities highlight functional divergence driven by subfamily-conserved residues, enabling each receptor to evolve a unique pharmacological profile. This functional divergence is underscored by the remarkable specificity of certain agonists for MRGPRX2. While MRGPRX1 and MRGPRX2 share high sequence identity, MRGPRX2 was activated by BAM 8-22 only with very low potency, much lower than its potency for activation of MRGPRX1. The broader MRGPR family shares several conserved motifs, yet each receptor exhibits a unique pharmacological fingerprint. The evolution of the MRGPR family likely reflects a trade-off between preserving core structural features required for G-protein coupling and receptor stability and allowing for specialization in ligand binding and signaling outputs. Residues that are conserved within the MRGPR subfamily but not across all Class A GPCRs may play pivotal roles in shaping these unique response profiles.

**Figure 9:**
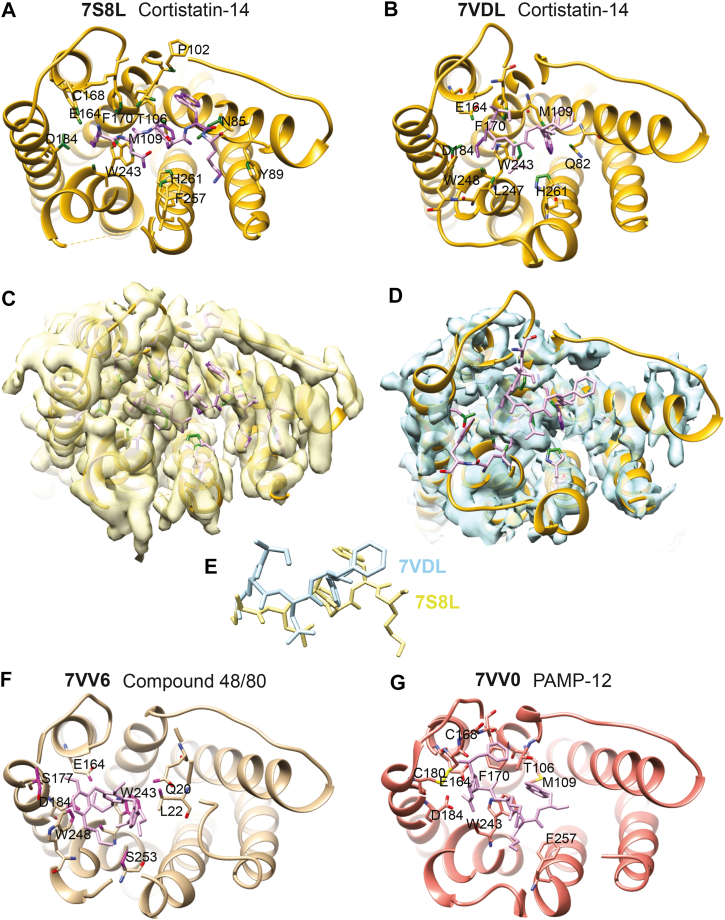
**Agonist binding site of MRGPRX2.***A* and *B*, models of cortistatin-14 (CS14) complexes with MRGPRX2 from two published cryo-EM-derived structures (*A*, PDB 7S8L (28); *B*, PDB 7VDL (35)). The receptor is depicted in gold, with atoms shown for the labeled residues that interact with CS14. Interacting atoms (distance < 3.8 Å) are colored *green*. The atoms modeled for CS14 are shown in lighter (*plum*) color, except for atoms interacting with MRGPRX2, colored *darker purple*. Note that the modeled CS14 residues are different for the two models: *A*, KNFFWK; *B*, CKNFF. There are only six interacting MRGPRX2 atoms in common between the two models. *C* and *D*, models are superimposed on the maps, with each threshold selected to display an included volume of 45,650 Å^3^. *E*, comparison of the two different poses for the ligand in 7S8L and 7VDL, oriented such that the receptor structures are aligned. *F* and *G*, models of MRGPRX2 complexes with compound 48/80 (*F*, PDB 7VV6) and PAMP-12 (*G*, 7VV0) (35).

The activation of MRGPRX2 by ligands with such diverse chemical structures is unusual among GPCRs. In attempting to understand the structural basis for the ligand responses observed, we examined published structures of ligand-MRGPRX2 bound to agonist compounds (28, 35). In general, ligand densities occupy a shallow cavity at the extracellular face of MRGPRX2, where side-chains are largely apolar except for negative charges of E164 and D184 (Fig. 9). However, for all ligands, only fragments of the structures, at most, were assigned to observed densities, with varying degrees of reliability. This partial resolution highlights the potential for structural plasticity within the MRGPRX2 ligand-binding pocket, which may contribute to its broad ligand selectivity. Residues lining the cavity are contributed primarily by the extracellular ends of TM helices 2, 5, 6, and 7, with some contributions from TM4 and from the TM4-5 and TM6-7 extracellular loops. These residues include I84(2.59 × 59), N85(2.60 × 6.0), V88(2.63 × 63), Y89(2.64 × 64), E164(4.60), F170(4–5 Loop), S177(5.33), D184(5.40), W243(6.55), F244(6.56 × 56), L247(6.59), W248(6.60), D254(6–7 Loop), F257(7.31 × 30), and H261(7.35 × 34). Relatively few consistent specific interactions with ligands can be identified in these structures, with most of the important interactions likely due to interactions with backbone atoms or with apolar side chains.

Understanding how different ligands interact with the receptor’s binding site is critical for elucidating its activation mechanisms. However, our results suggest that mutations located at the cytoplasmic face or toward the cytoplasmic end of TM helices, sites well removed from the binding site(s) at or near the extracellular face, can also affect potency. As the ligands tested are either known through structures or inferred from membrane impermeability to act at residues facing the extracellular space, the effects of mutations at or near the cytoplasmic face are likely mediated by allosteric coupling between the cytoplasmic effector-interacting loops and the ligand binding site(s) (reviewed in (68)).

Different ligands likely induce slightly different conformational pathways to activation, consistent with the differential sensitivity of ligands to amino acid substitutions that we observed. For example, L31V had deleterious consequences for vancomycin, atracurium, and compound 48/80, but appeared to have no deleterious effect on the potency of ciprofloxacin or polymyxin B. I277T had a significant effect only on activation by atracurium.

Many MRGPRX2 ligands are low-potency agonists. However, for drugs administered intravenously, by injection, or topically, transient local concentrations can be substantially higher than circulating plasma levels. For example, vancomycin is injected as a solution or administered as eye drops at a concentration >3 mM and up to 16.8 mM (69, 70). Vancomycin is well known to trigger the so-called vancomycin infusion reaction, mediated by MRGPRX2-dependent mast cell degranulation (40). Because vancomycin has been extensively studied in this context, clinically observed exposure levels provide a useful physiological reference point for evaluating the concentrations used in our assays.

The goal of this study was to systematically compare the ability of multiple ligands to activate MRGPRX2 variants in a controlled assay platform. Comparison with physiological mast cell responses to some of the same ligands supports the suitability of our assay for comparing the potency of ligands at different receptor variants. MRGPRX2-dependent calcium release and degranulation have been observed in human peripheral blood-derived cultured mast cells in response to atracurium and ciprofloxacin, with pEC50s in a similar range (3.4 for atracurium, compared to 4.0 in our study; 3.5 for ciprofloxacin, compared to 4.0 in our study) (49). LAD-2 cells, which express endogenous MRGPRX2, or rat basal leukemia (RBL) cells stably transfected with MRGPRX2, degranulated in response to vancomycin with pEC50s of 3.5 and 3.6, respectively (compared to 3.7 in our study) and to substance P with pEC50s of 5.3 and 5.5, respectively (compared to 6.0 in our study) (71). The fact that our observed pEC50 values, while similar, are mostly higher than those for mast cell degranulation, suggests that overexpression in HEK cells does not lead to “spare receptors” as compared to the physiological situation in mast cells, perhaps because much of the overexpressed protein is retained in internal membranes. However, it is possible that some mutations may affect receptor function or engagement of downstream effectors differently in mast cells than in HEK cells. While the experiments here exclusively employed G_αq/11_-mediated Ca^2+^ mobilization, physiologically, MRGPRX2 may couple to additional G proteins, such as G_αi_ (14, 27, 28), and to additional downstream pathways. It will be interesting in the future to characterize these interactions, and the differential effects of ligands and mutations on them.

**Figure 10:**
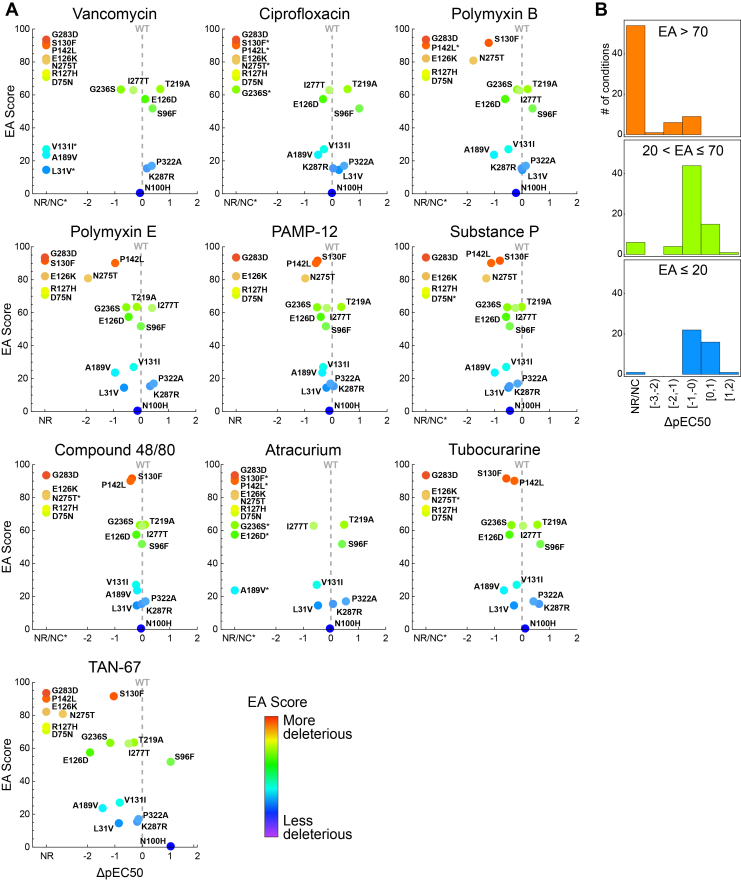
**Relationship between EA scores and changes in pEC50.***A*, dot plots show the EA scores plotted against the change in pEC50 relative to WT MRGPRX2 for each variant. Each dot represents an individual mutant, and colors correspond to EA scores, with a gradient indicating predicted functional impact. Zero is indicated by a dotted *gray line* for reference. Variants for which pEC50 values could not be calculated are shown at the *left*. *B*, histogram of NR/NC or ΔpEC50 values for all drugs combined and mutants pooled according to EA score (EA > 70, 20 < EA ≤ 70, and EA ≤ 20). The proportion of non-measurable values (NR or NC) for mutants with EA > 70 was significantly different from the proportion of non-measurable values in the other two tiers (chi-squared test, *p* < 0.0001). “# of conditions” refers to the number of combinations of mutation and ligand in the plotted ΔpEC50.

EA scores were reasonably good predictors of a substitution’s functional impact (Fig. 10). The two mutations that resulted in lack of expression and/or complete lack of responsiveness to all ligands, E126K and G283D, had EA scores >80. Low-scoring substitutions were more likely to have measurable EC50s, while high-scoring substitutions were more likely to have no response. The only case of a low-scoring substitution with no response was A189V (EA score = 24) with vancomycin, and L31V (EA 15) and V131I (EA 27) were NC for atracurium and/or vancomycin. The distribution of non-measurable responses and ΔpEC50s for all drugs are shown in Figure 10*B*. For variants with EA>70, the primary phenotype was loss of pEC50 measurement capability, consistent with severe loss of function. For mutants with moderate EA scores (20 < EA ≤ 70), there were dramatically fewer NR/NC cases, with most conditions resulting in measurable negative ΔpEC50s. The lowest scoring variants (EA ≤ 20) primarily fell in a near-symmetric distribution of ΔpEC50s around zero, with almost complete disappearance of NR/NC cases.

The varied responses of individuals to MRGPRX2 agonists, leading to DHRs in some cases, suggest the presence of gain-of-function alleles in the population. Recently, several gain-of-function missense mutations resulting from SNPs have been identified. Naturally occurring S325L and L329Q, near the C-terminus, demonstrated apparently enhanced response to substance P in transfected RBL cells (27). Three variants identified from patients with DHRs to quinolones or vancomycin, N16H, N62S, and S313R, had enhanced responses to one or more of vancomycin, ciprofloxacin, or substance P (31). Interestingly, N62S and N16H were also identified in a cohort of chronic spontaneous urticaria patients, but only N62S was enriched compared to the population; RBL cells expressing N62H exhibited enhanced degranulation and Ca^2+^ mobilization in response to substance P (32). All the previously identified gain-of-function variants have moderate EA scores: N16H (EA 21), N62S (EA 37), S313R (EA 40), S325L (EA 85), and L329Q (EA 31).

We also identified four variants resulting in increased potency of at least one ligand. S96 is located at the extracellular end of TM2 (Fig. 8*E*). Although this residue does not contact the parts of ligands visible in MRGPRX2 structures (28, 35), it could influence access to or the shape of the ligand binding cavity. K287 is near the cytoplasmic end of TM7, and P322 is in the C-terminal domain; both of these could potentially affect G protein binding. Finally, T219 is at the cytoplasmic end of TM6, with the side chain facing out to solvent and not contacting the bound G protein. Gain-of-function variants at these residues all have low to moderate EA scores: S96F (EA 52), T219A (65), K287R (EA 15), P322A (EA 17). High EA scores indicate rare substitutions at evolutionarily important positions, which likely experienced negative selection. However, variants leading to gain of function for agonism by exogenous ligands would not necessarily be deleterious in physiological conditions. Consistent with this, none of these substitutions had a significant effect on the potency of the two endogenous ligands tested, substance P and PAMP-12. These results suggest that SNPs with moderately scoring missense mutations might be a productive pool from which to identify gain-of-function variants associated with DHRs.

## Experimental procedures

### Cell lines and growth conditions

HEK293 wildtype (WT) and ΔG_q/11_ knockout cells (39, 72) were kindly gifted by Asuka Inoue (Tohoku University) and maintained in Dulbecco’s modified Eagle’s medium (Corning) supplemented with 10% fetal bovine serum (Sigma) and 1% Penicillin-Streptomycin (Gibco). Cells were grown in a 37 °C humidified incubator with 5% CO_2_.

### Expression constructs

WT human MRGPRX2 with an N-terminal hemagglutinin signal sequence (“HAss”) followed by a FLAG tag was PCR amplified from the MRGPRX2 TANGO plasmid (73) (Addgene plasmid 66440), adding a stop codon following the MRGPRX2 coding sequence. The insert was cloned into pcDNA3.1; the final construct (pcDNA3.1-HAss-FLAG-MRGPRX2) included the HAss FLAG tag but not the C-terminal TEV cleavage site and transcription factor-coding sequence from the TANGO plasmid. Mutants were constructed from pcDNA3.1-HAss-FLAG-MRGPRX2 by site-directed mutagenesis. Empty pcDNA3.1 was used as the empty vector (EV) control for all experiments. Human G_αq_ was constructed from mouse G_αqo_ (a gift from Bruce Conklin, UCSF), containing an internal HA tag replacing G_αq_ amino acids 125 to 130, by site-directed mutagenesis to substitute S171A, producing humanized G_αqo_, then replacing the C-terminal amino acids of the chimera with the corresponding residues from G_αq_. All constructs were verified by Sanger sequencing.

### Calcium mobilization assay

40,000 HEK293 cells per well were seeded on black clear-bottom poly-d-lysine–coated 96-well plates (Corning BioCoat). Approximately 6 h later, cells were transfected with 50 ng/well of DNA (pcDNA3.1-HAss-FLAG-MRGPRX2 WT or mutant, or EV) using Lipofectamine 2000 (Invitrogen) according to the manufacturer’s instructions. Assays were carried out ∼40 h after transfection. Cells were washed one time with 100 μl KRH buffer (10 mM HEPES, 4.7 mM KCl, 2.2 mM CaCl_2_, 1.2 mM KH_2_PO_4_, 1.2 mM MgSO_4_, 120 mM NaCl and 1.8*g*/L glucose, pH 7.4, supplemented with 1 mM probenecid (TCI, P1975) to inhibit export of Fluo-4). Next, cells were loaded with 50 μl 1.7 μM Fluo-4 AM (Invitrogen, F14201) in KRH with 0.01% Pluronic F-127 (VWR, 59004) for 1 h at room temperature in the dark. Cells were washed once with 100 μl KRH, then 100 μl of KRH was added to each well. The assay plate and drug plate containing 3X ligand dilutions in KRH and 4X ATP (200 μM) were pre-incubated for 10 min in a plate reader (Flex Station 3, Molecular Devices) that was preheated to 37 °C. 50 μl of 3X ligand was added at 20 s and 50 μl of 4X ATP was added at 110 s. The presence of an ATP response was used as a positive control for live cells. When ΔG_αq_ cells were used, ionomycin (5 μM) was used instead of ATP. Measurements were acquired every ∼2 s (Excitation at 488 nm, Emission at 520 nm). All Ca^2+^ mobilization assays were conducted with three technical replicates per condition and included WT and EV controls on the same plate, and assays were repeated on at least three separate days. Data from each well were baseline-corrected by subtracting the average of the first 12 data points. No additional transformations or normalizations were performed. The peak value within the time interval from 20 to 60s (for C48/80 20 to 40s was used) was determined for each concentration of agonist, and then the average of the peak value together with the previous and following data points was used to generate a dose-response curve. Estimates of EC50 were taken from the least-squares best fit of a sigmoidal dose response to log(agonist) vs peak amplitudes, assuming a single binding site (Hill coefficient = 1). Curve fitting was performed in Graphpad Prism v8 or v5, and the negative log_10_ of the EC50, pEC50, was derived from the dose-response curve.

### Immunofluorescence for surface expression determination

80,000 HEK293 cells were seeded on poly-d-lysine coated coverslips in 24-well plates. Approximately 6 h later, cells were transfected with 800 ng of DNA (pcDNA3.1-HAss-FLAG-MRGPRX2 WT or mutant, or EV) per well using Lipofectamine 2000 (Invitrogen) according to the manufacturer’s instructions. Approximately 40 h post-transfection, cells were fixed with 2% PFA in PBS for 10 min, then blocked for 15 min in PBS with 1% BSA (non-permeabilizing) or PBS with 1% BSA and 0.1% Triton X-100 (permeabilizing). Cells were labeled with FLAG antibody (mouse monoclonal M2, Millipore Sigma) diluted 1:500 in blocking buffer for 45 min, followed by Alexa-488-conjugated goat anti-mouse (Invitrogen) 2 ug/ml for 30 min. Coverslips were mounted with Prolong Diamond (Invitrogen). Images were acquired with a Zeiss LSM710 or a Leica TCS-SP5 confocal microscope using a 63× oil immersion objective. Alexa-488 and DAPI were detected sequentially using a 488 nm Argon laser and 405 nm diode laser, respectively, for excitation. 1024 × 1024 pixel (132 nm/pixel) 8 bit single optical sections were acquired, taking care to avoid saturated pixels. Four to eight images were acquired for each coverslip. All samples in a given permeabilization group were imaged with identical microscope settings. Intensity in the Alexa-488 channel was quantified using Mathematica v.13 (Wolfram) as described previously (74). Raw images were first subjected to thresholding to remove values in the bottom 2%, and then total intensity was determined. Values were normalized by dividing by the average of the WT determinations from the same day, and the mean of the technical replicates is reported.

### Ligands

Ligands used were vancomycin hydrochloride (Santa Cruz catalog no. 1404-93-9), ciprofloxacin hydrochloride (Sigma catalog no. PHR1044), polymyxin B sulfate salt (Sigma catalog no. P4932), polymyxin E (colistin, Santa Cruz catalog number sc-204696), PAMP-12 (Fisher catalog no. 65-511), substance P (Sigma catalog no. 05-23-06000), BAM8-22 trifluoroacetate salt (Sigma catalog no. SML0729), chloroquine diphosphate salt (Sigma catalog no. C6628), compound 48/80 (Sigma catalog no. C2313), poly-l-lysine (Sigma catalog no. P0879), atracurium Besylate (Santa Cruz catalog no. 64228-81-5), tubocurarine (Tocris catalog no. 2820/50), TAN-67 (SB 205607, Fisher 09-211), Leucine Enkephalin acetate salt hydrate (Sigma catalog no. L9133). Ciprofloxacin and chloroquine were made fresh before every experiment by dissolving first in PBS as 10 mM solutions. All other ligands were dissolved in PBS as 10 mM or 100 mM stock solutions and stored in aliquots at −20 °C. Before every experiment, all ligands were further diluted in KRH; final ligand concentrations in assay wells were 10^−9^, 10^−8^, 10^−7^, 10^−6^, 10^−5^, 10^−4^, or 10^−3^ M, except 10^−3^ M was not tested for BAM 8-22 or Substance P. For polydisperse polymers, accurate molar concentrations could not be calculated; final concentrations of poly-l-lysine were 0.03, 0.1, 0.3, 1, 3, 10, and 30 μg/ml, and final concentrations of Compound 48/80 were 0.003, 0.01, 0.03, 0.1, 0.3, 1, and 3 μg/ml. Using a degree of polymerization between 3 and 6 for Compound 48/80, as reported by the manufacturer, this corresponds to a range of ∼3.3 × 10^−9^−6.5 × 10^−9^ M to ∼3.3 × 10^−6^−6.5 × 10^−6^ M Compound 48/80.

## Data availability

All data are included in the Figures and Supporting Material. Materials are available upon request.

## Supporting information

This article contains supporting information.

## Conflict of interest

The authors declare that they have no conflicts of interest with the contents of this article.
