# Supplementary material for: Structural determinants of ligand response specificity in the mast cell activating GPCR, MRGPRX2
Source: J Biol Chem. 2026 May 8;302(6):113132. doi: 10.1016/j.jbc.2026.113132 (PMC13266011; doi:10.1016/j.jbc.2026.113132)
Supplement: Supplementary Figures [file mmc1.pdf]

Figure S1

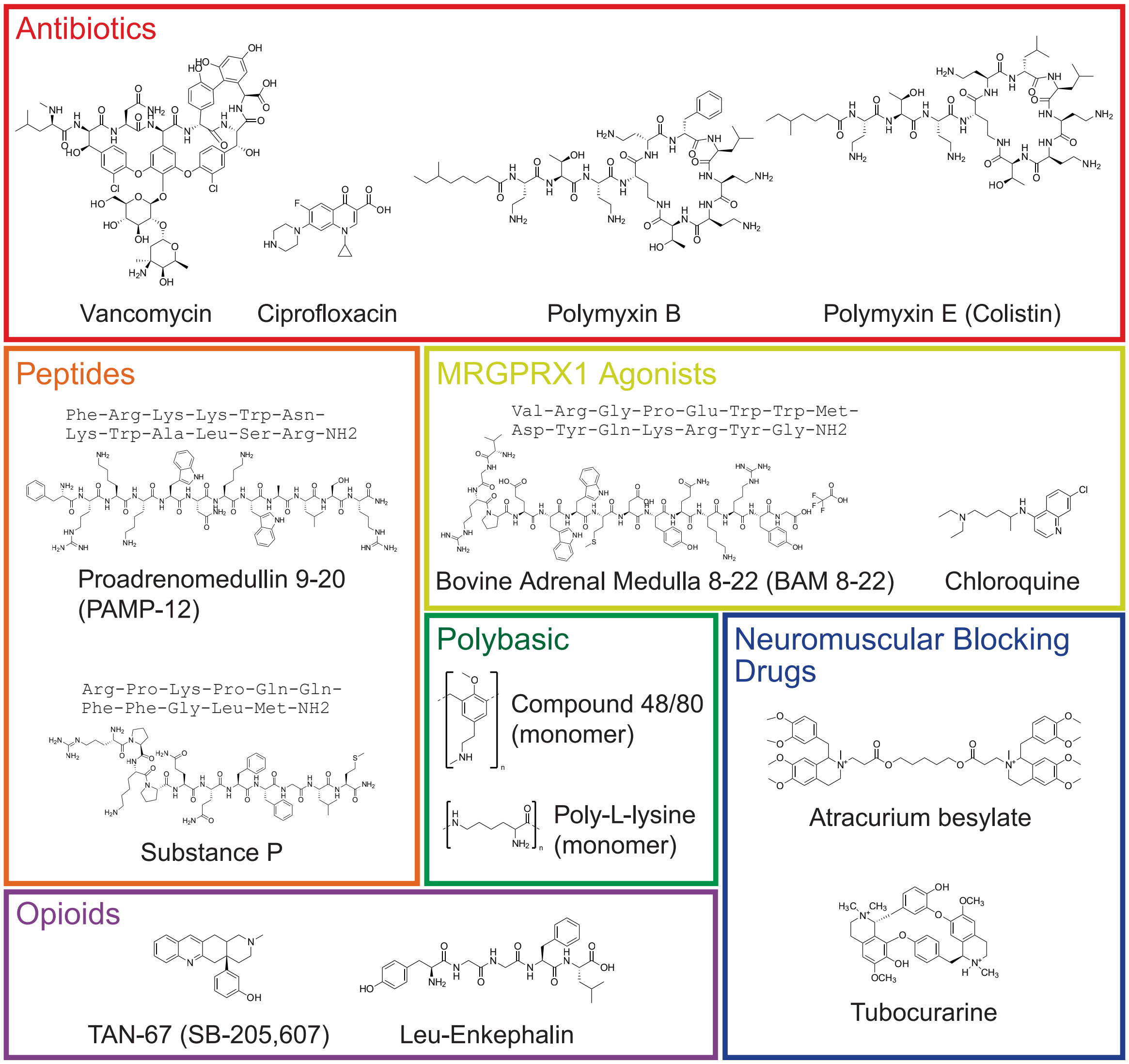


**Figure S1. Ligands used in experiments.** Molecules representing a variety of agonists that have been reported to be either mast cell activators, MRGPRX2 agonists, or agonists for MRGPRX1 (a closely related member of the MRGPRX subfamily of Class A GPCRs).

# Figure S2

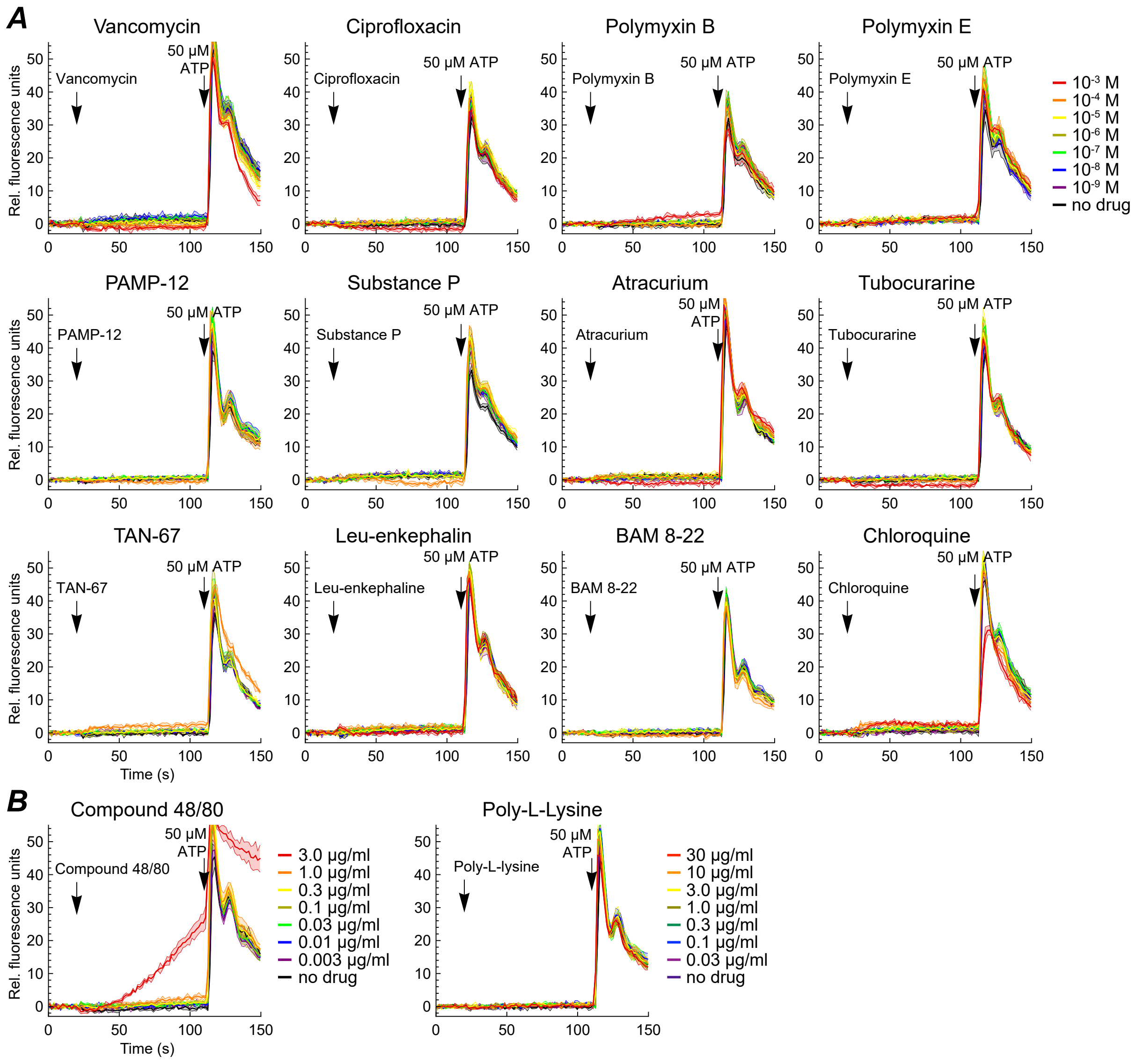


**Figure S2. Empty vector controls validating specificity of MRGPRX2 response to various agonists in HEK cells.** Representative $Ca^{2+}$ mobilization traces showing intracellular calcium responses of HEK cells transiently transfected with empty vector (pcDNA 3.1) and stimulated with various compounds. Arrows indicate injection of agonist at 20 s, followed by injection of 50 μM ATP at 110 s as a positive control. Shaded regions indicate s.d. of technical replicates.

# Figure S3

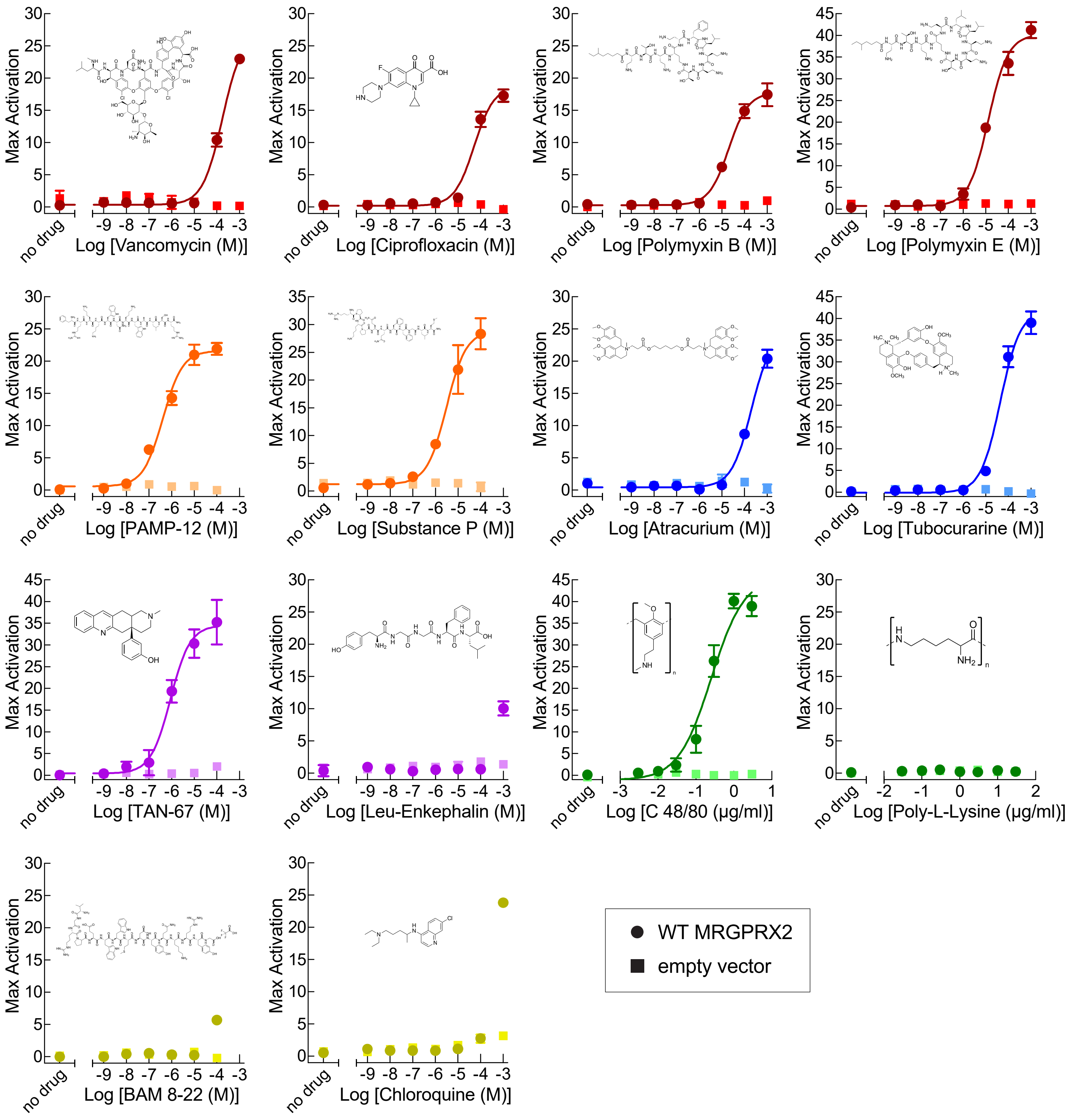


**Figure S3. Representative dose response curves of WT MGPRX2 and empty vector control in response to various ligands.** Dose-response curves were generated by plotting the peak fluorescence intensity in response to each concentration of ligand. Points and error bars represent the mean ± SD of three technical replicates.

Figure S4

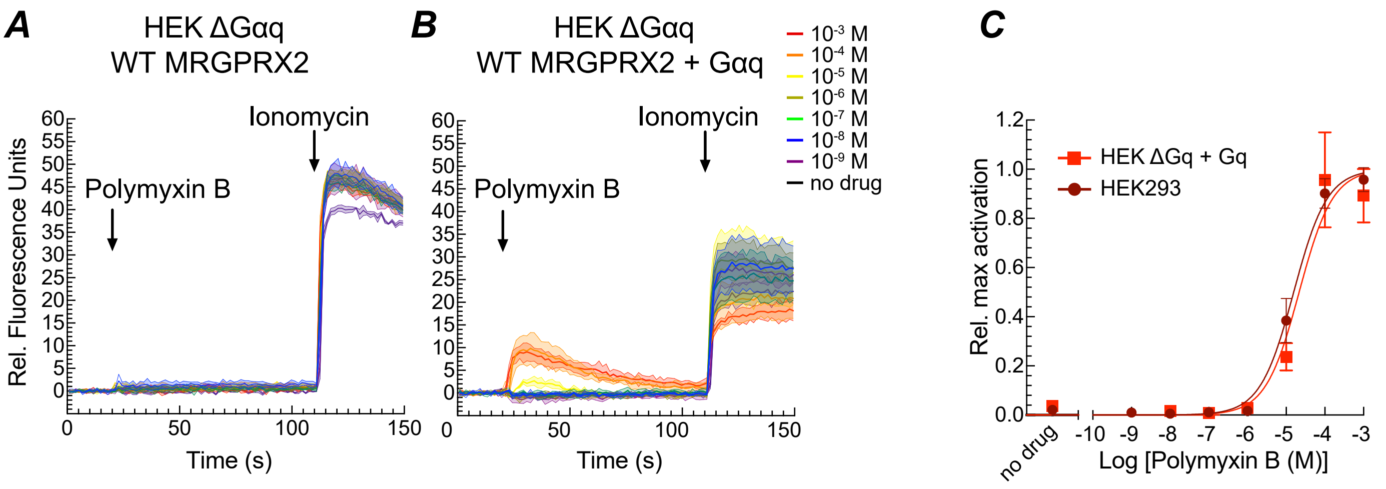


**Figure S4. MRGPRX2 response to polymyxin B is Gq dependent.** (**A, B**) Representative $Ca^{2+}$ mobilization traces from HEK ΔGαq knockout cells transfected with WT MRGPRX2 (**A)** or co-transfected with WT MRGPRX2 and Gαq (**B**). Ionomycin was injected at 110 s as a positive control. (**C**) Dose response curves generated from data as in **A** and **B**. Curves are shown normalized to top and bottom plateaux to highlight potency comparison.

Figure S5

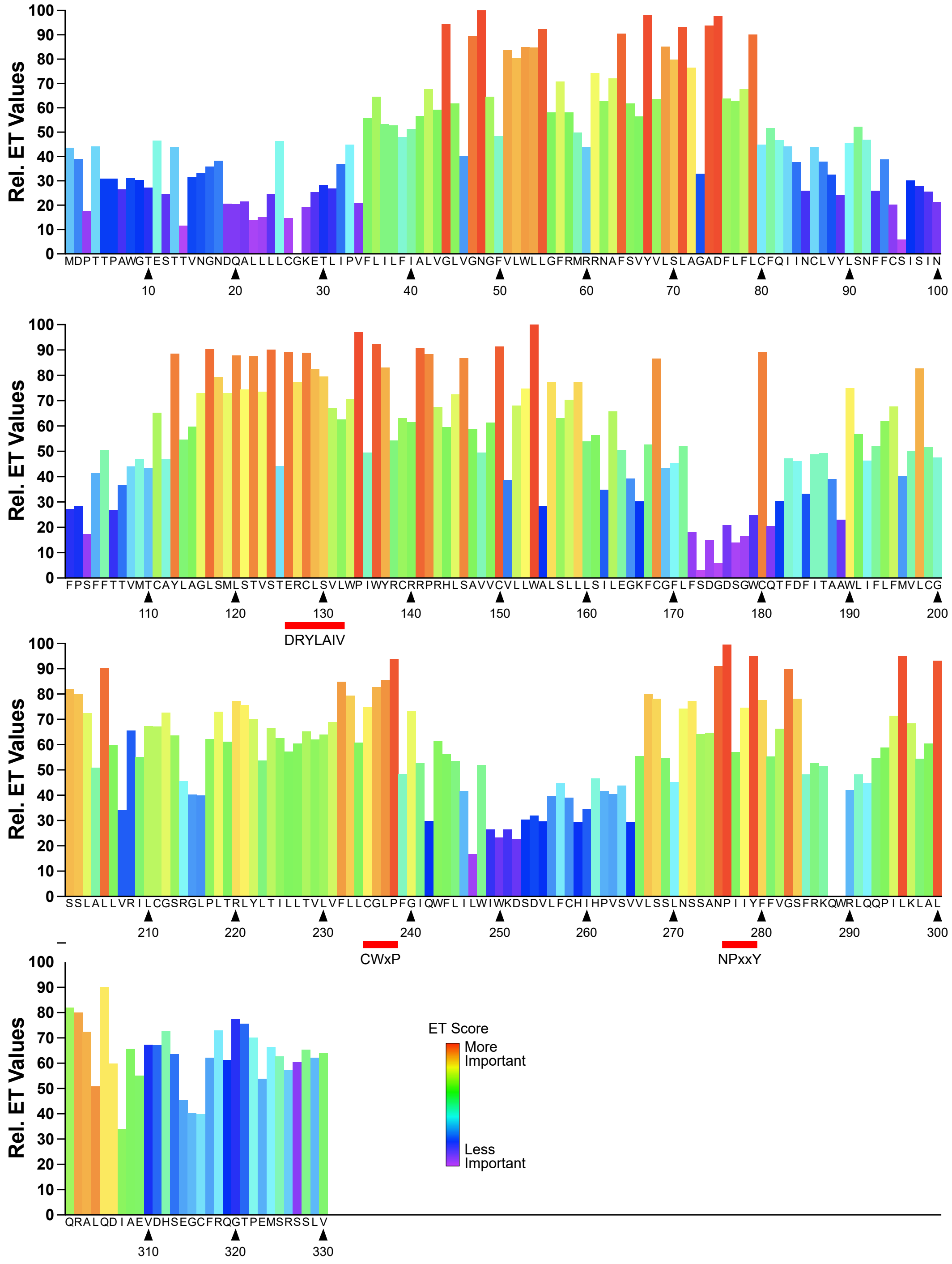


**Figure S5. Evolutionary Trace (ET) analysis of MRGPRX2 residues.** Bar graph showing ET scores (ranging from 0 to 100) for each amino acid position in the MRGPRX2 sequence.

Figure S6

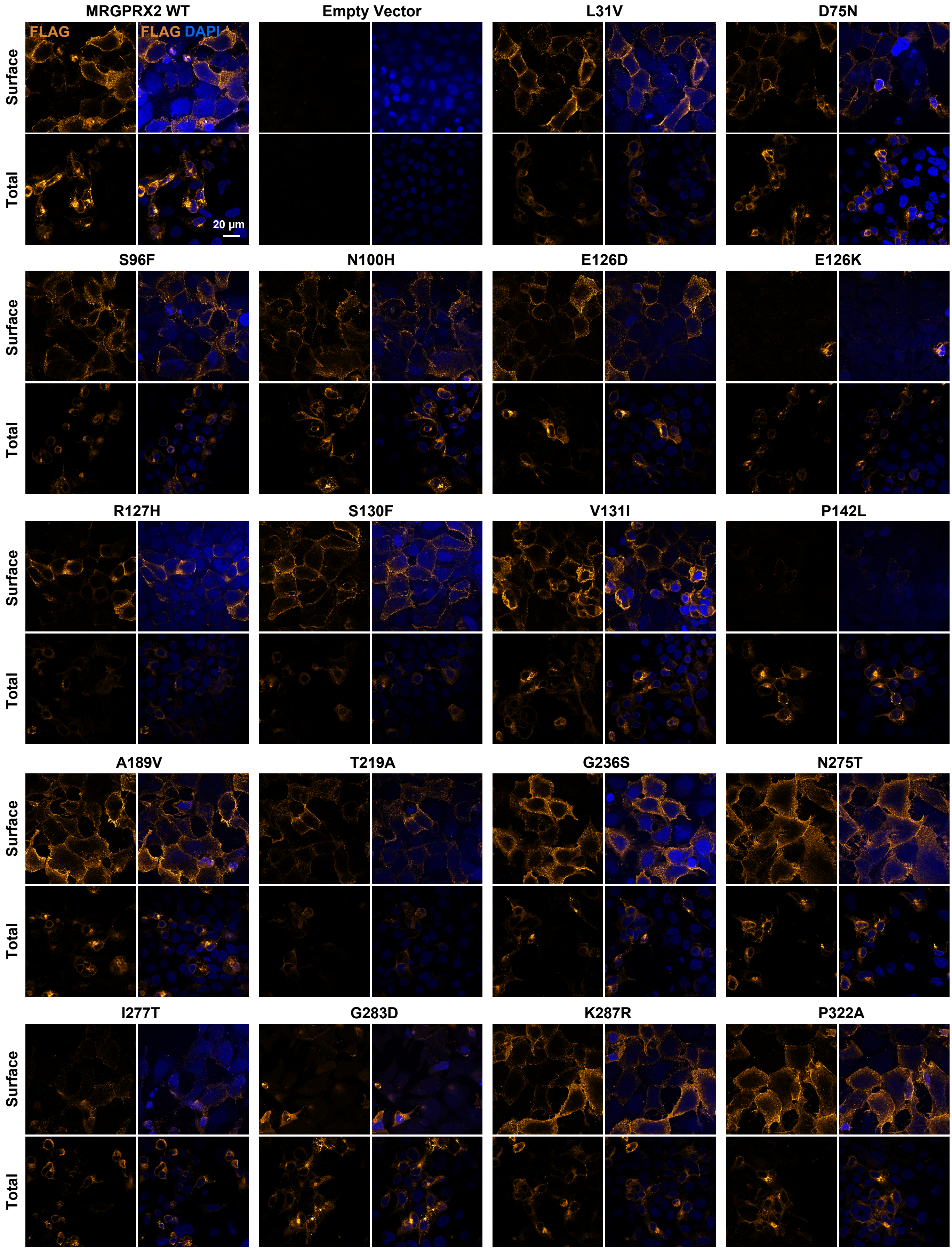


**Figure S6. Surface expression of MRGPRX2 variants.** Representative images of transfected HEK cells immunostained with anti-FLAG antibody (orange) in non-permeabilizing conditions to detect surface MRGPRX2 or in permeabilizing conditions to detect total MRGPRX2.

Figure S7

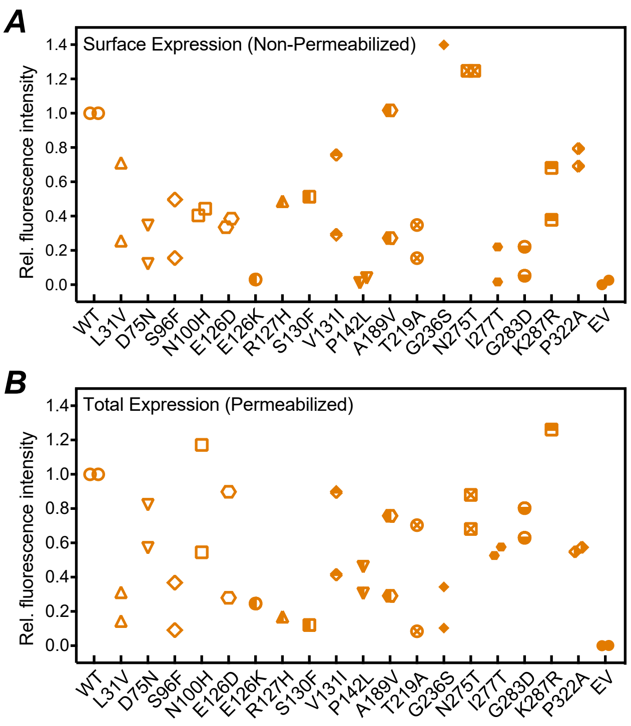


**Figure S7. Relative expression of MRGPRX2 variants.** Relative surface (**A**) and total (**B**) expression quantified from labeling intensity in images as shown in Figure S6. Each point represents the mean of 4-8 technical replicate images.

Figure S8

**Figure S8. Activation of MRGPRX2 variants in response to different ligands.** Representative $Ca^{2+}$ mobilization traces for HEK cells transfected with WT or variant MRGPRX2, or empty vector (EV), and stimulated with (**A**) vancomycin, (**B**) ciprofloxacin, (**C**) polymyxin B, (**D**) polymyxin E, (**E**) PAMP-12, (**F**) substance P, (**G**) BAM 8-22, (**H**) chloroquine, (**I**) compound 48/80, (**J**) poly-L-lysine, (**K**) atracurium, (**L**) tubocurarine, (**M**) TAN-67, or (**N**) leu-enkephalin. Arrows indicate injection of drug at 20 s, followed by injection of 50 μM ATP at 110 s as a positive control. Shaded regions indicate s.d. of technical replicates. EV plots are duplicated from Fig. S2.

**A**

***Vancomycin***

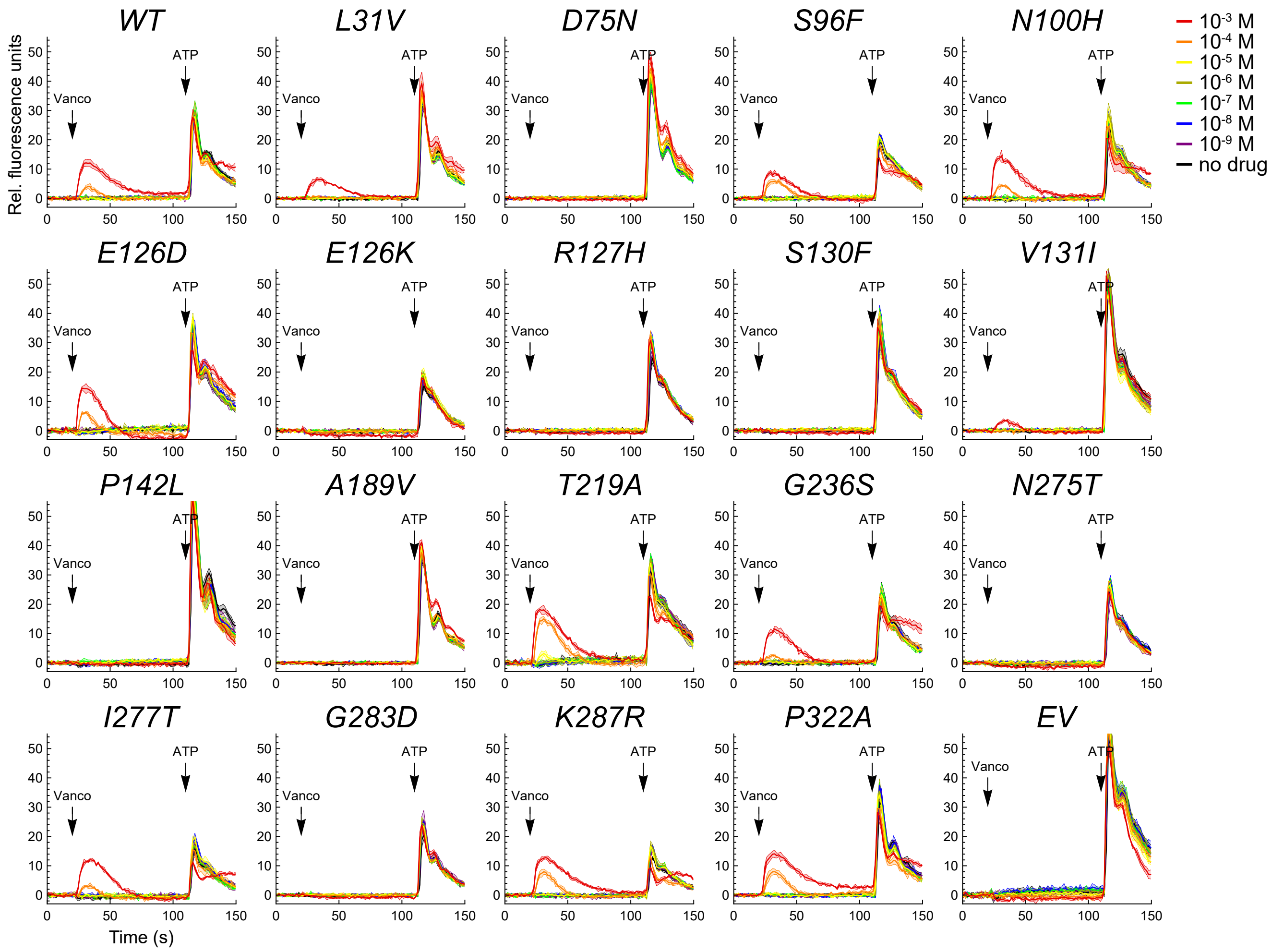

# Figure S8

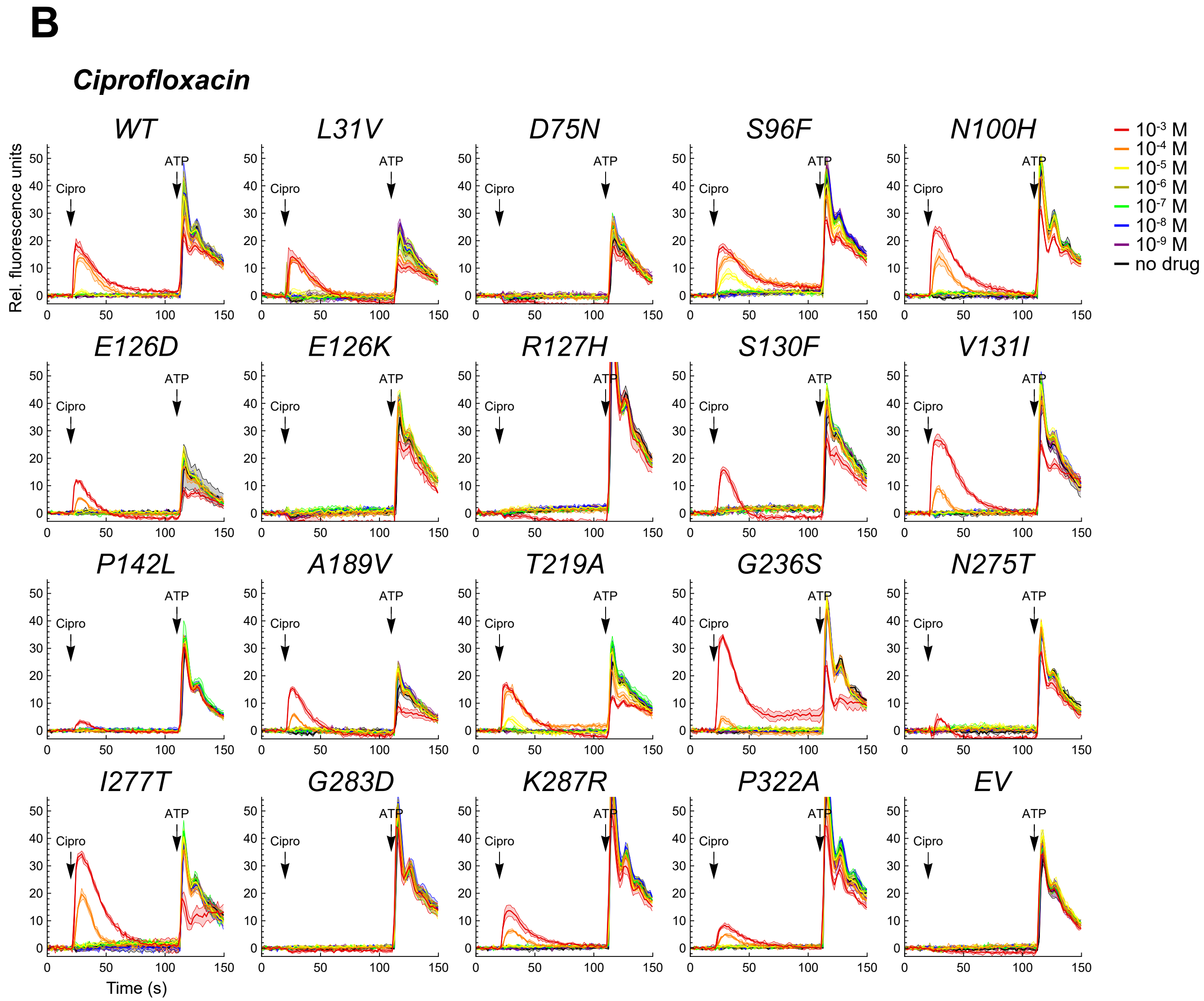
B
Ciprofloxacin
WT
L31V
D75N
S96F
N100H
E126D
E126K
R127H
S130F
V131I
P142L
A189V
T219A
G236S
N275T
I277T
G283D
K287R
P322A
EV
Cipro
ATP
Rel. fluorescence units
Time (s)
10-3 M
10-4 M
10-5 M
10-6 M
10-7 M
10-8 M
10-9 M
no drug

Figure S8

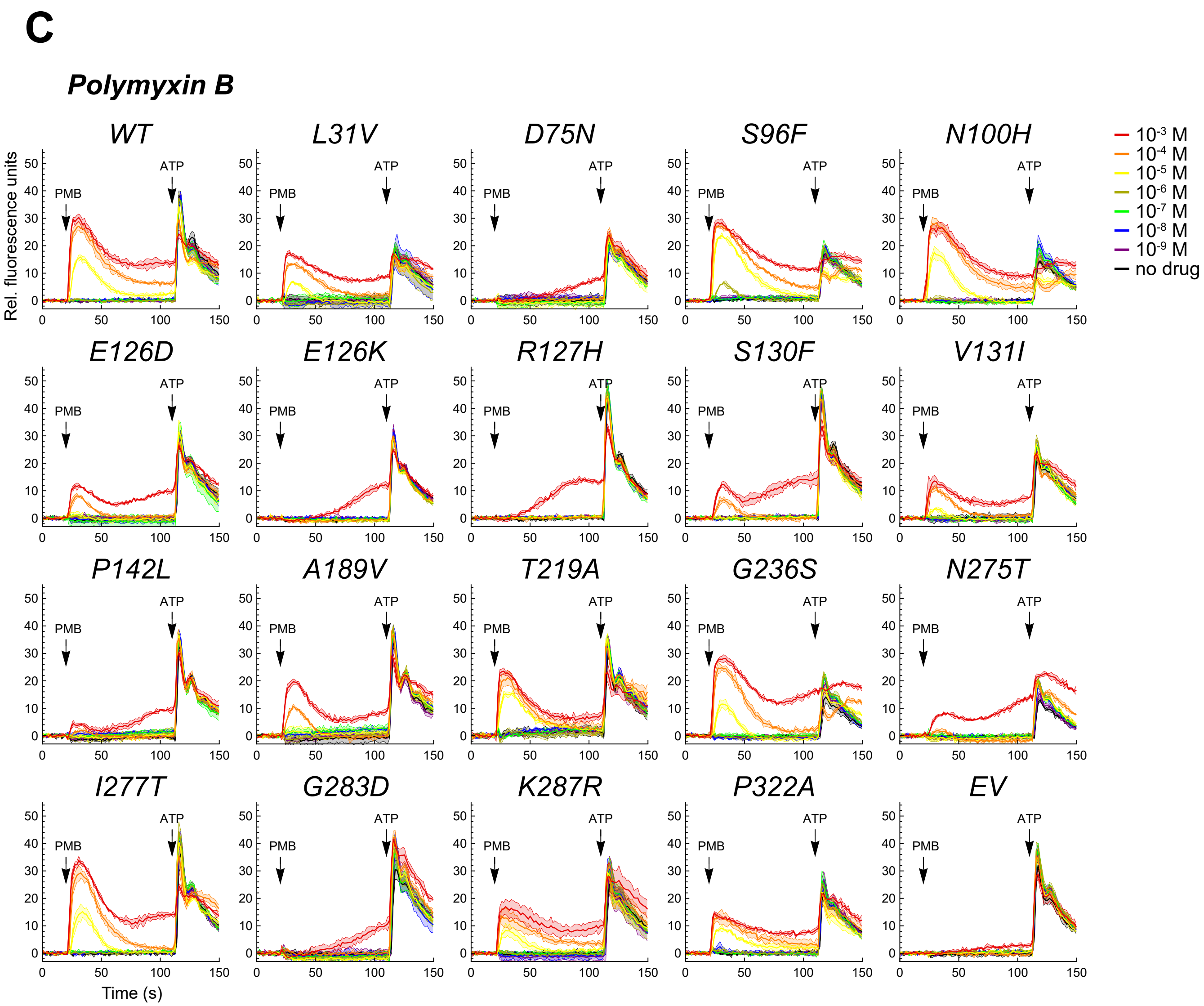
C
Polymyxin B
WT
L31V
D75N
S96F
N100H
E126D
E126K
R127H
S130F
V131I
P142L
A189V
T219A
G236S
N275T
I277T
G283D
K287R
P322A
EV
PMB
ATP
Rel. fluorescence units
Time (s)
10-3 M
10-4 M
10-5 M
10-6 M
10-7 M
10-8 M
10-9 M
no drug

Figure S8

# D

***Polymyxin E***

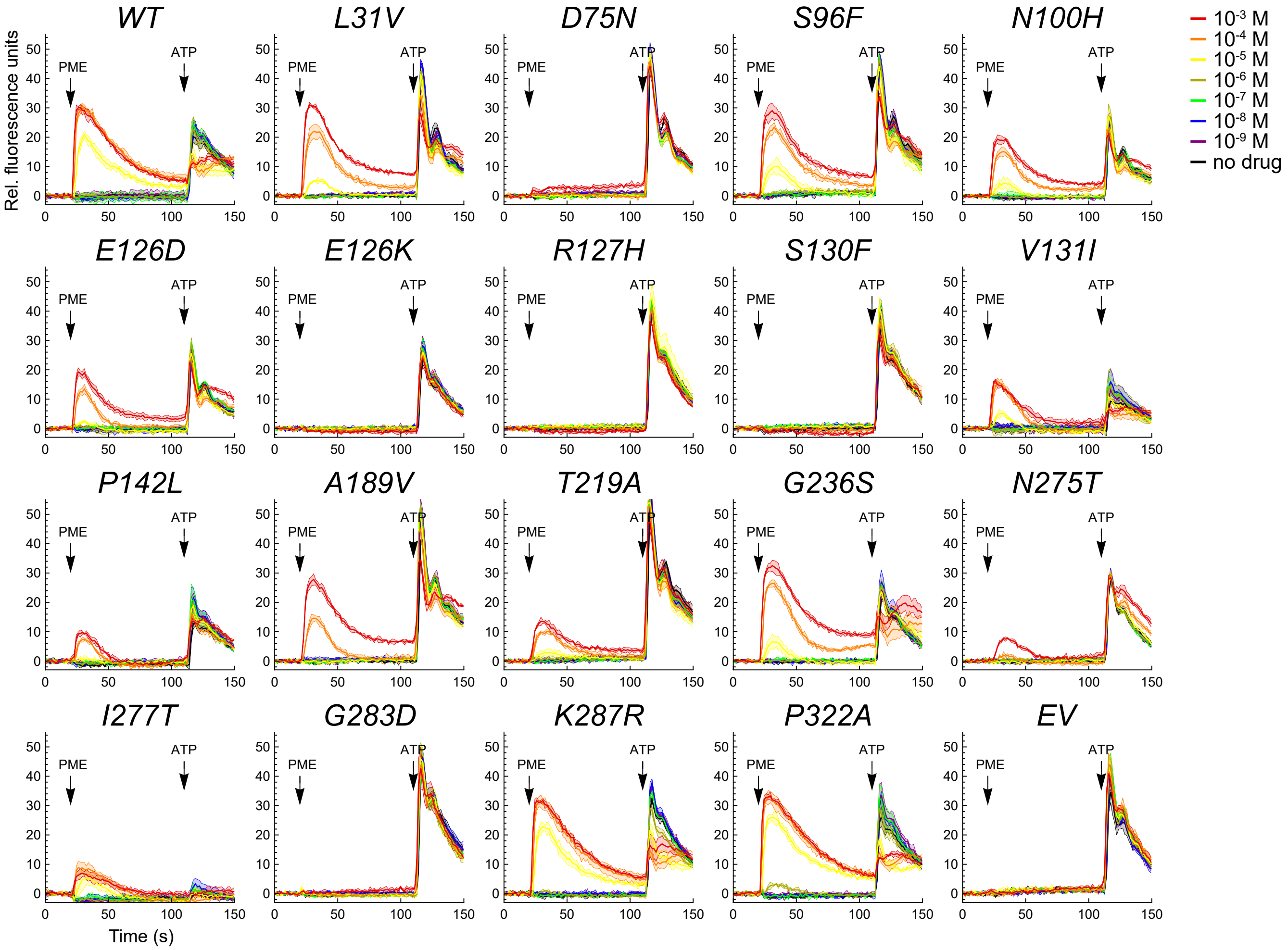

Figure S8

# E

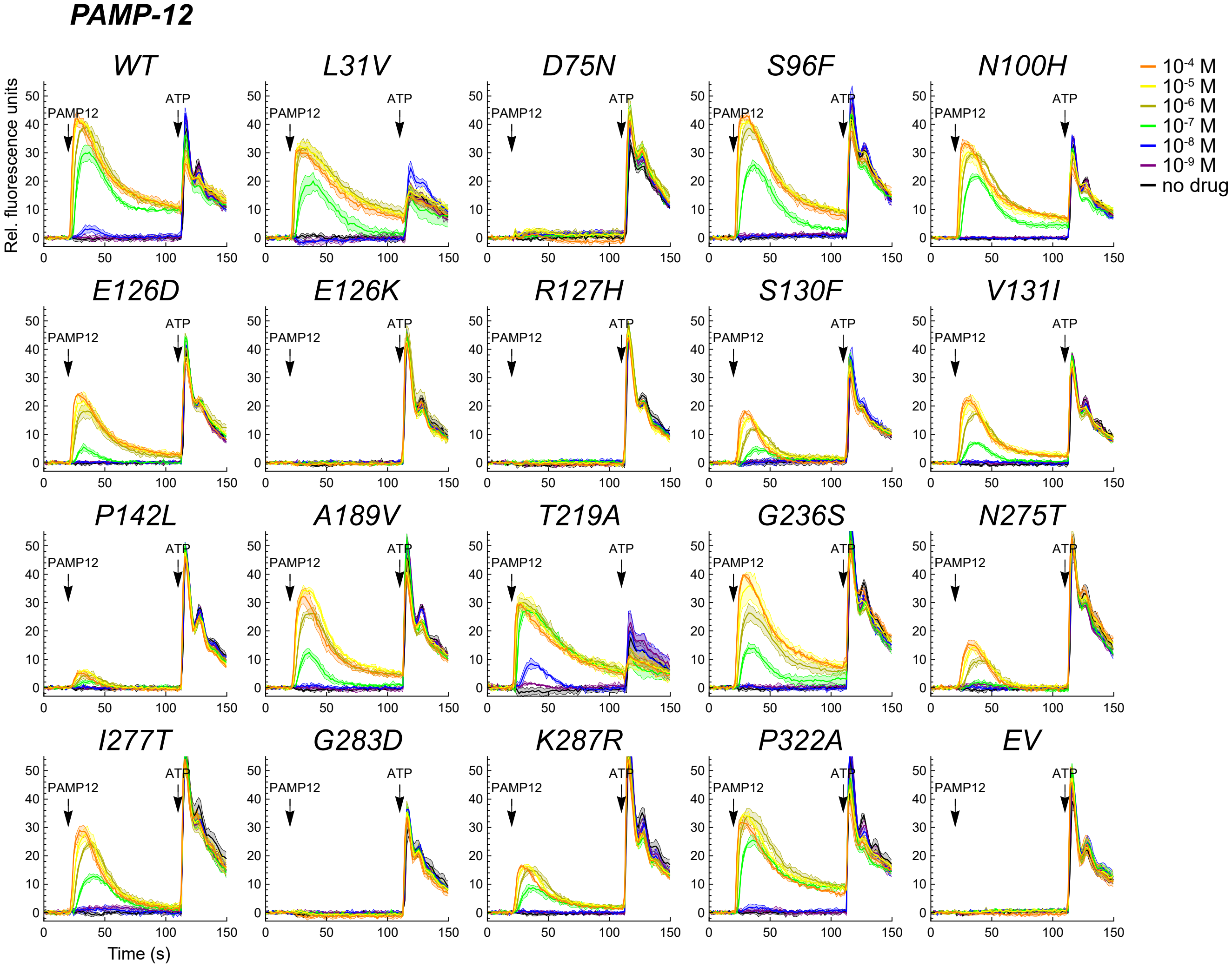
PAMP-12
WT
L31V
D75N
S96F
N100H
E126D
E126K
R127H
S130F
V131I
P142L
A189V
T219A
G236S
N275T
I277T
G283D
K287R
P322A
EV
PAMP12
ATP
Rel. fluorescence units
Time (s)
10-4 M
10-5 M
10-6 M
10-7 M
10-8 M
10-9 M
no drug

Figure S8

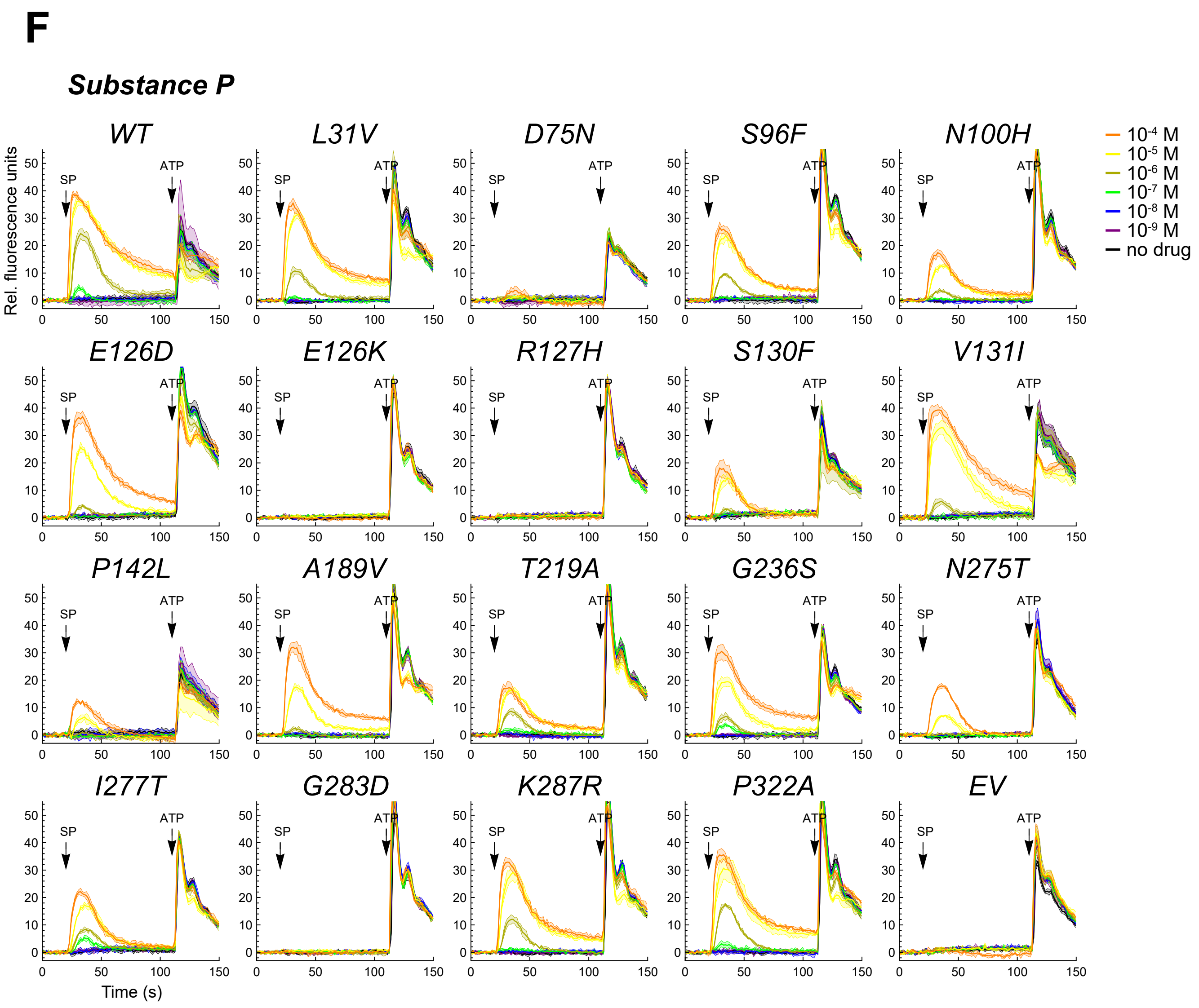

F
Substance P
WT
L31V
D75N
S96F
N100H
E126D
E126K
R127H
S130F
V131I
P142L
A189V
T219A
G236S
N275T
I277T
G283D
K287R
P322A
EV
SP
ATP
Rel. fluorescence units
Time (s)
10-4 M
10-5 M
10-6 M
10-7 M
10-8 M
10-9 M
no drug

Figure S8

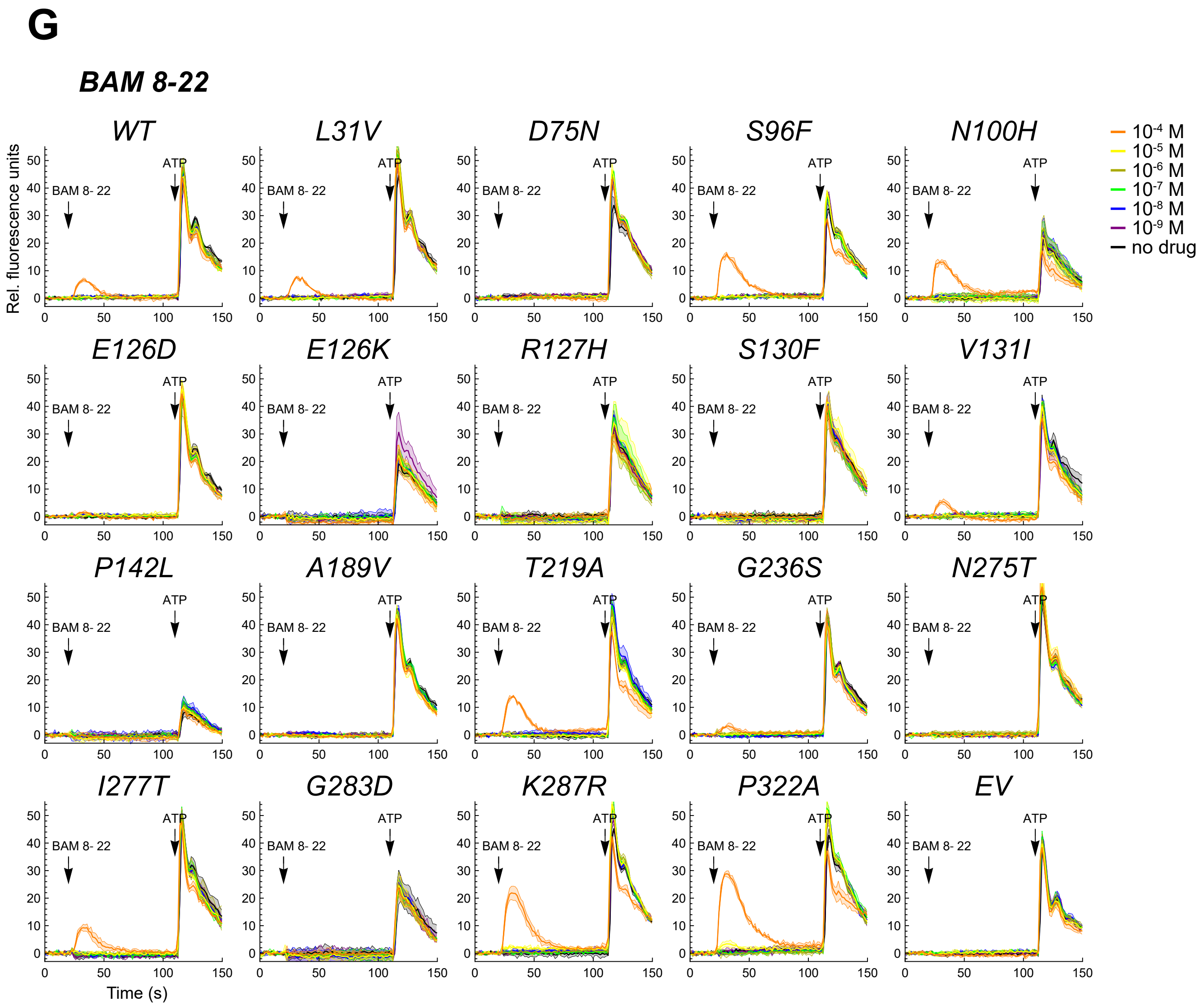
G
BAM 8-22
WT
L31V
D75N
S96F
N100H
E126D
E126K
R127H
S130F
V131I
P142L
A189V
T219A
G236S
N275T
I277T
G283D
K287R
P322A
EV
BAM 8- 22
ATP
Rel. fluorescence units
Time (s)
10-4 M
10-5 M
10-6 M
10-7 M
10-8 M
10-9 M
no drug

Figure S8

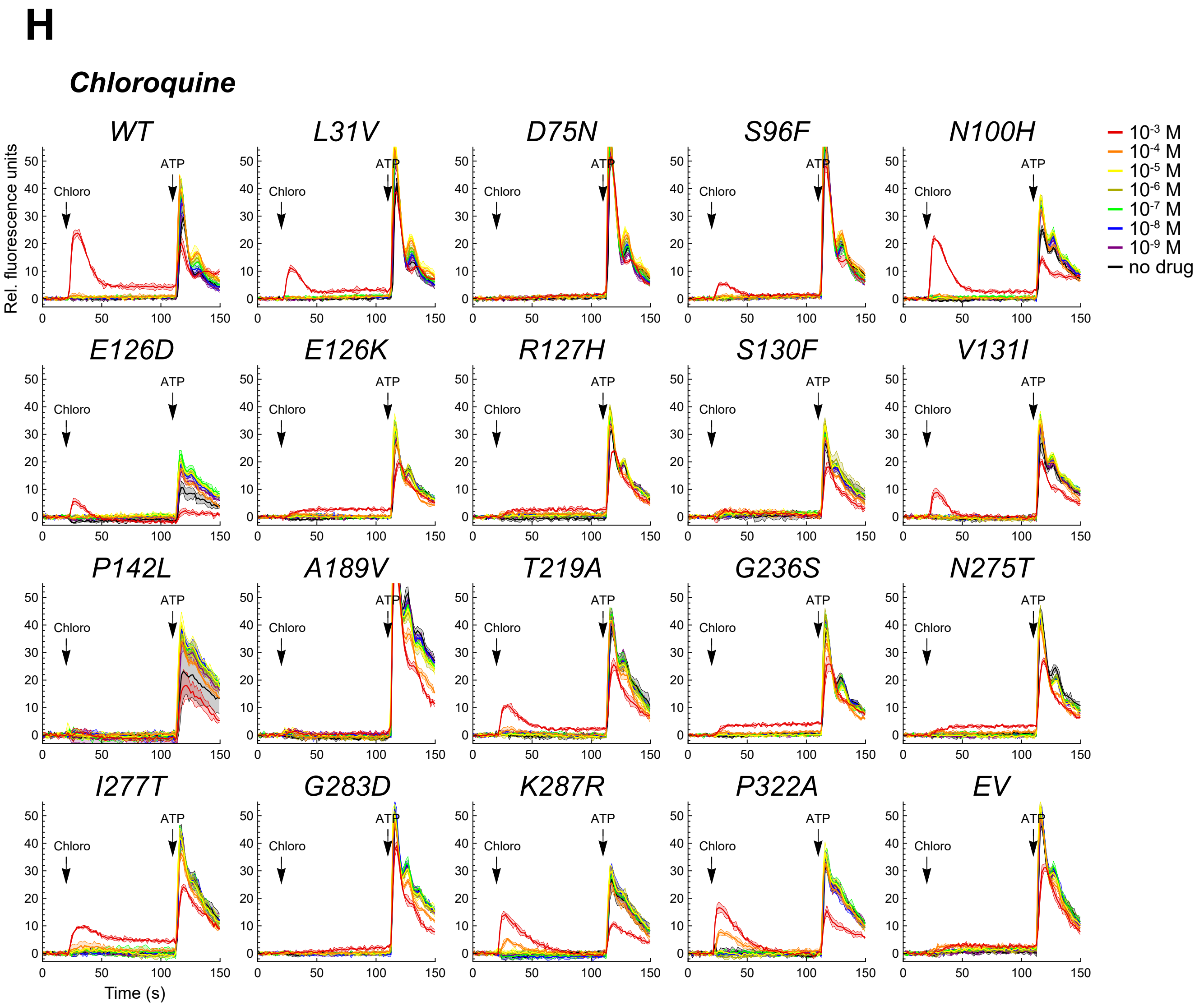
H
Chloroquine
WT
L31V
D75N
S96F
N100H
E126D
E126K
R127H
S130F
V131I
P142L
A189V
T219A
G236S
N275T
I277T
G283D
K287R
P322A
EV
Chloro
ATP
Rel. fluorescence units
Time (s)
10-3 M
10-4 M
10-5 M
10-6 M
10-7 M
10-8 M
10-9 M
no drug

# Figure S8

## I

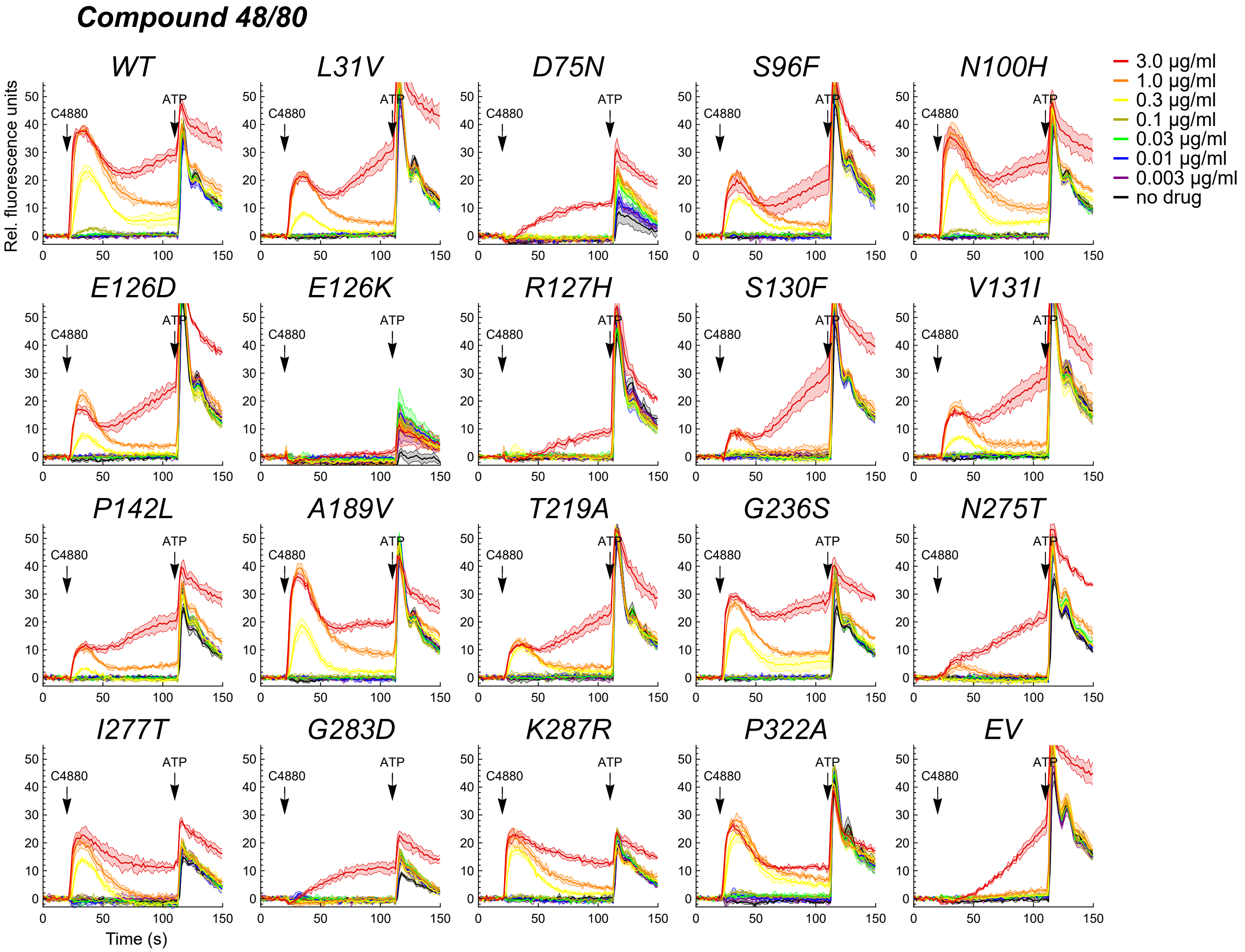

Compound 48/80
WT
L31V
D75N
S96F
N100H
E126D
E126K
R127H
S130F
V131I
P142L
A189V
T219A
G236S
N275T
I277T
G283D
K287R
P322A
EV
C4880
ATP
Rel. fluorescence units
Time (s)
3.0 µg/ml
1.0 µg/ml
0.3 µg/ml
0.1 µg/ml
0.03 µg/ml
0.01 µg/ml
0.003 µg/ml
no drug

Figure S8

# J

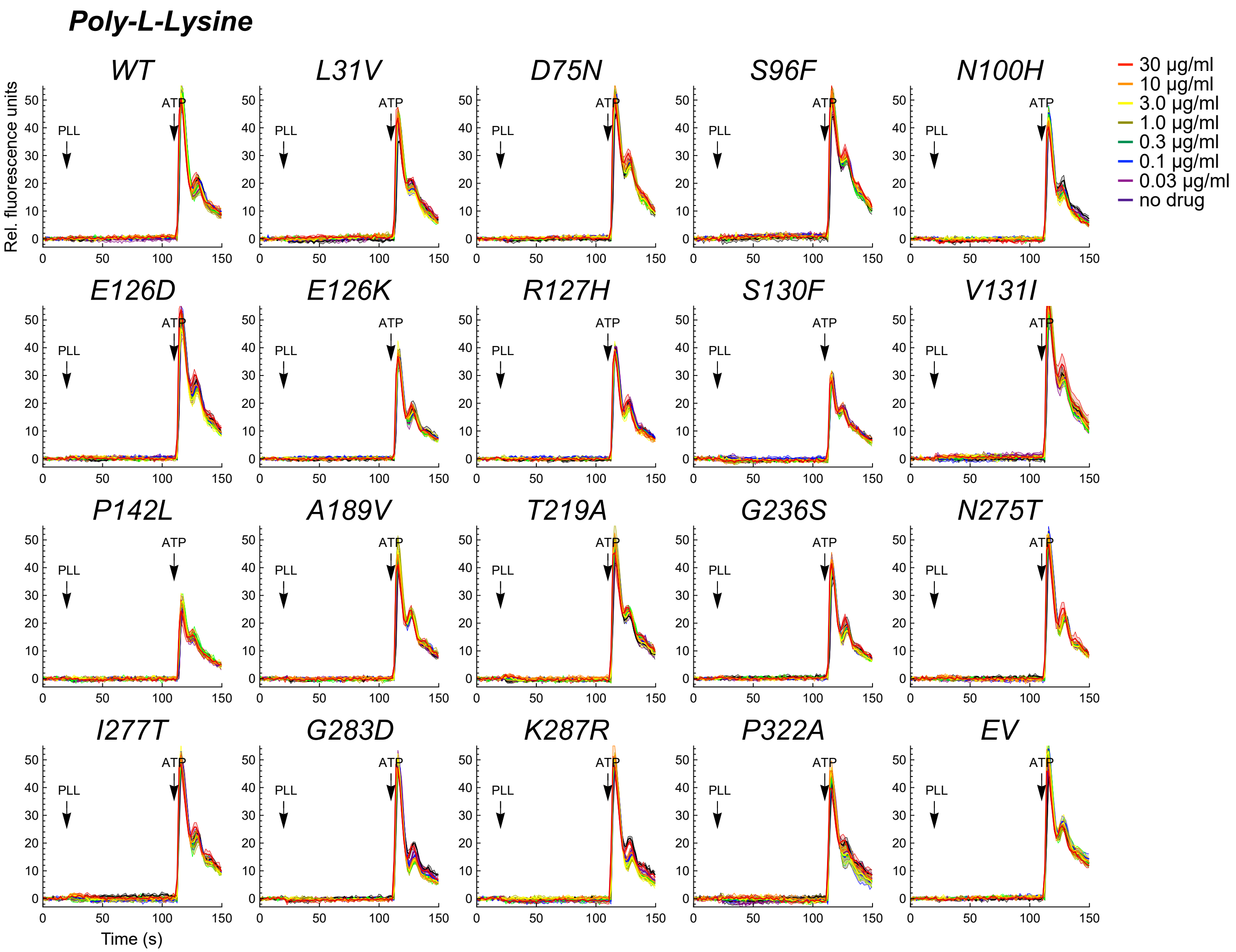

Poly-L-Lysine
WT
L31V
D75N
S96F
N100H
E126D
E126K
R127H
S130F
V131I
P142L
A189V
T219A
G236S
N275T
I277T
G283D
K287R
P322A
EV
PLL
ATP
Rel. fluorescence units
Time (s)
30 μg/ml
10 μg/ml
3.0 μg/ml
1.0 μg/ml
0.3 μg/ml
0.1 μg/ml
0.03 μg/ml
no drug

# Figure S8

## K

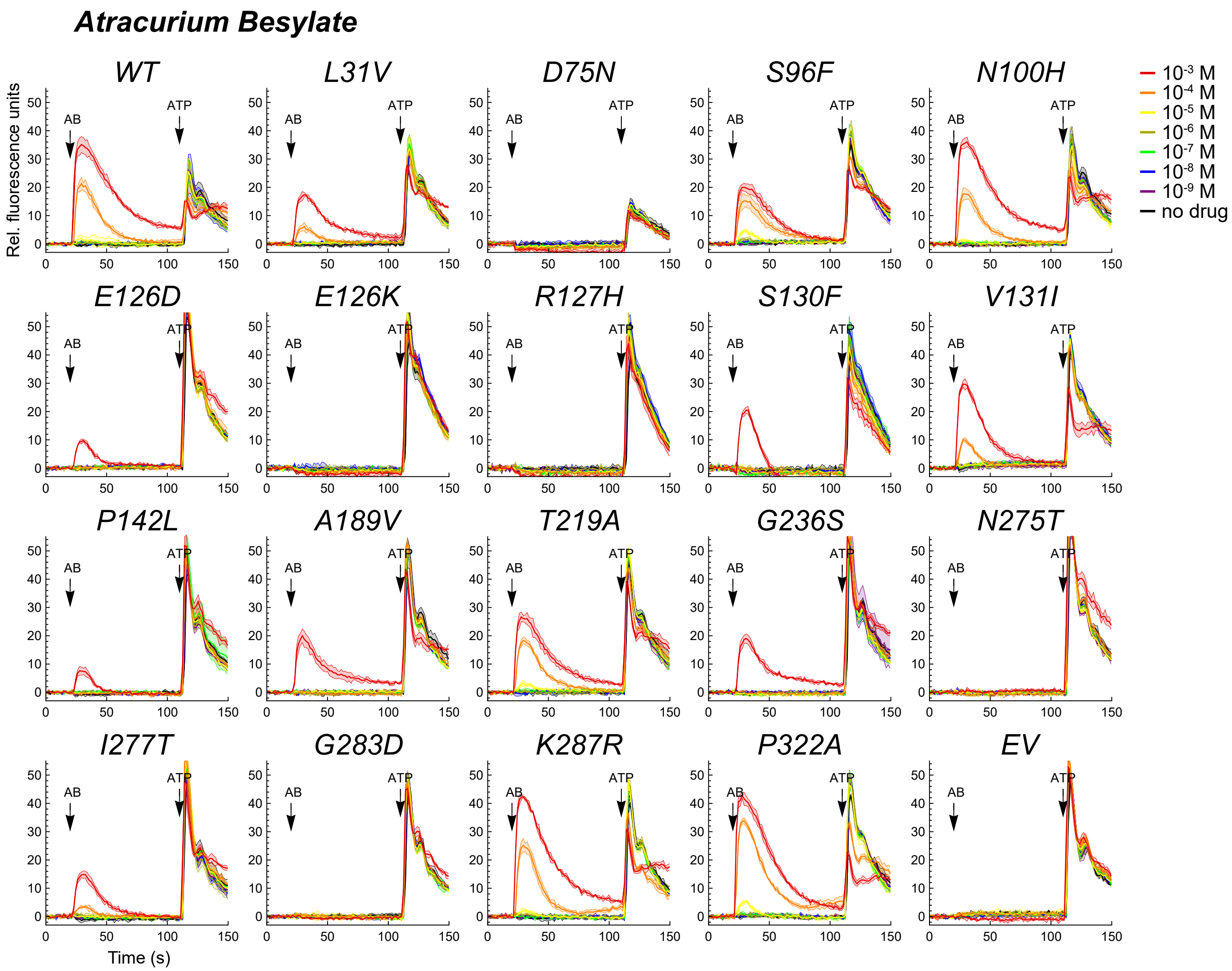

Atracurium Besylate
WT
L31V
D75N
S96F
N100H
E126D
E126K
R127H
S130F
V131I
P142L
A189V
T219A
G236S
N275T
I277T
G283D
K287R
P322A
EV
AB
ATP
Rel. fluorescence units
Time (s)
0
50
100
150
10
20
30
40
10-3 M
10-4 M
10-5 M
10-6 M
10-7 M
10-8 M
10-9 M
no drug

# Figure S8

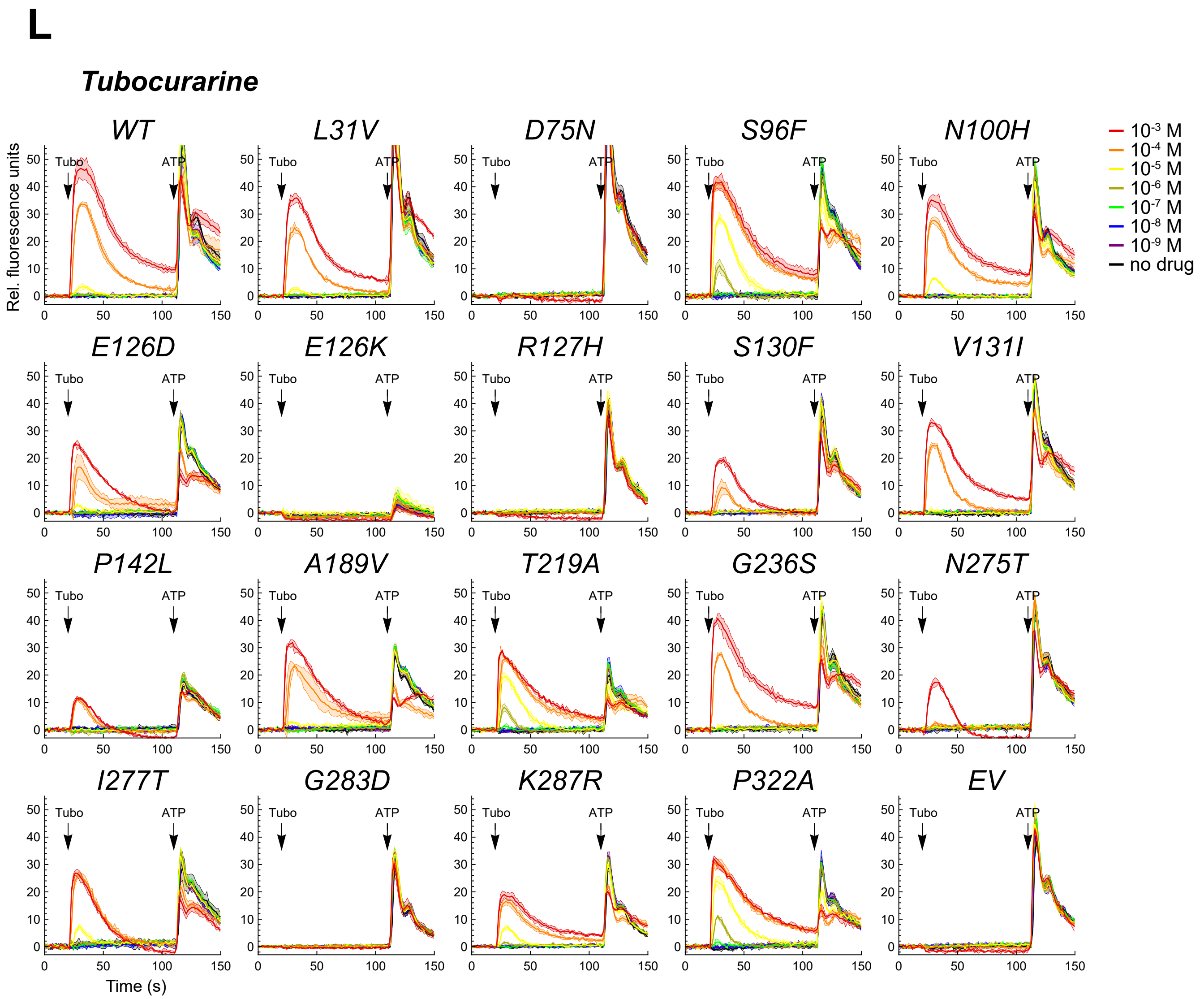

L
Tubocurarine
WT
L31V
D75N
S96F
N100H
E126D
E126K
R127H
S130F
V131I
P142L
A189V
T219A
G236S
N275T
I277T
G283D
K287R
P322A
EV
Tubo
ATP
Rel. fluorescence units
Time (s)
10-3 M
10-4 M
10-5 M
10-6 M
10-7 M
10-8 M
10-9 M
no drug

# Figure S8

## M

***TAN-67***

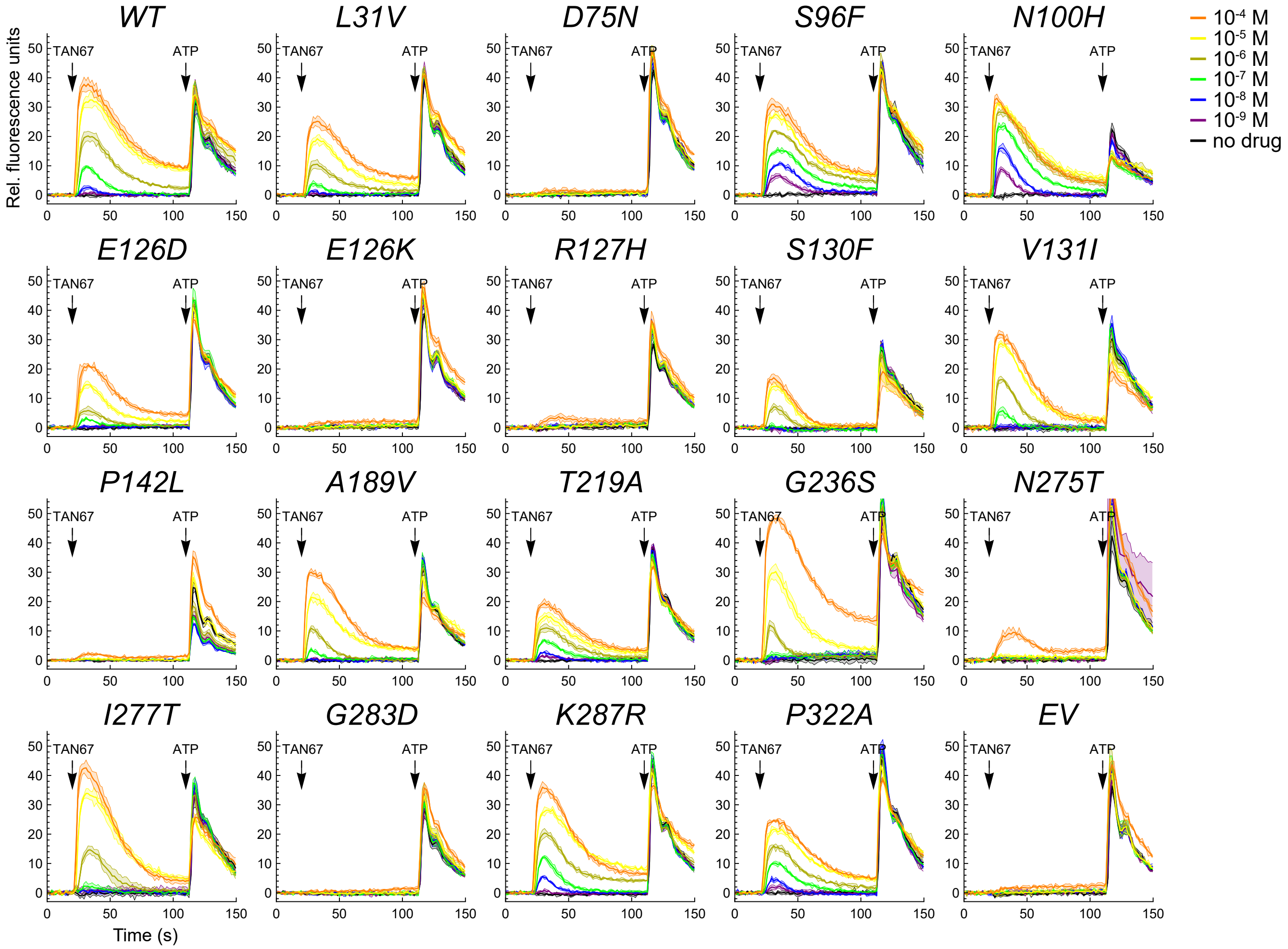

Figure S8

**N**

***Leu-Enkephalin***

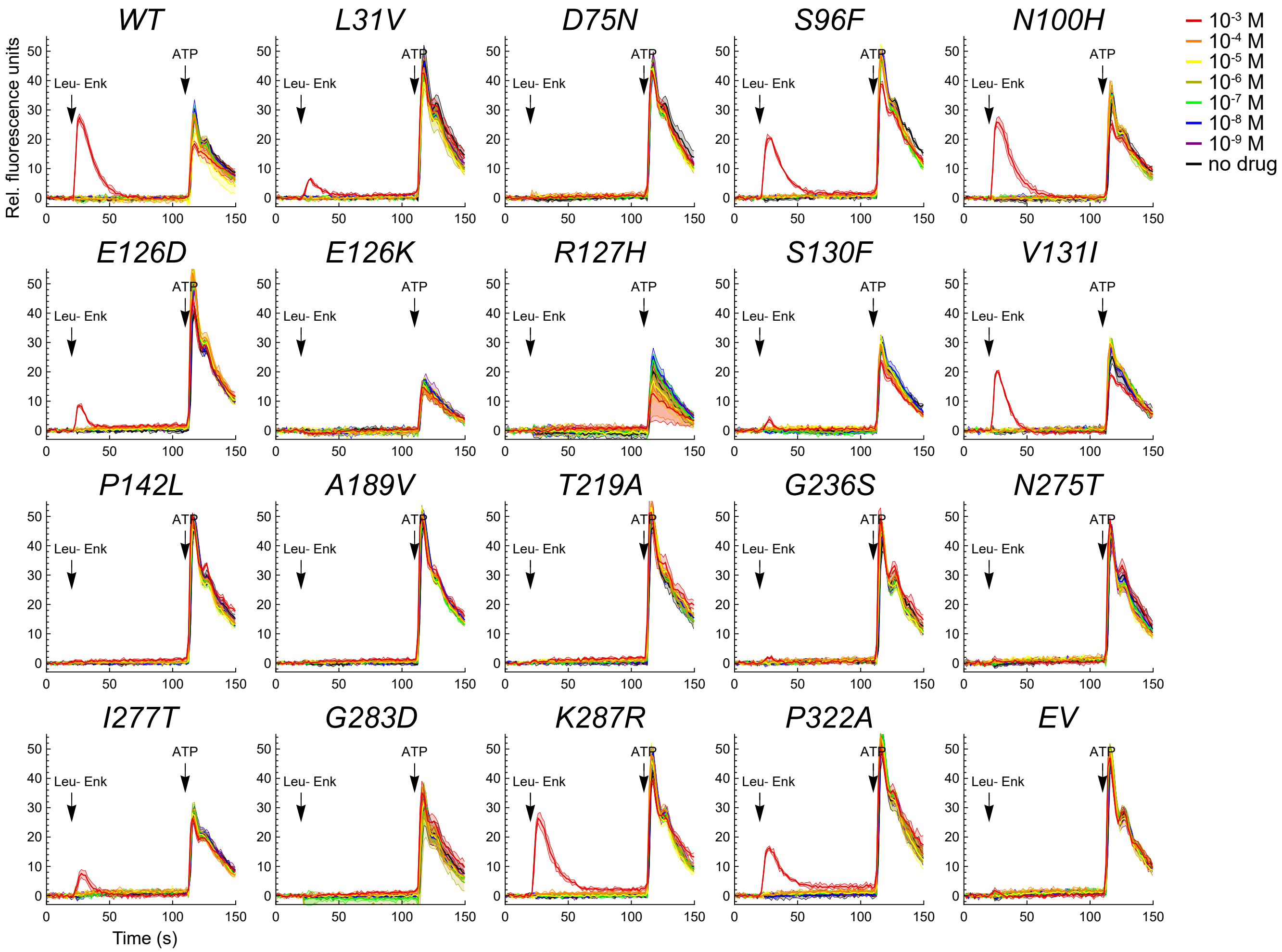


**Figure S8. Activation of MRGPRX2 variants in response to different ligands.** Representative $Ca^{2+}$ mobilization traces for HEK cells transfected with WT or variant MRGPRX2, or empty vector (EV), and stimulated with (**A**) vancomycin, (**B**) ciprofloxacin, (**C**) polymyxin B, (**D**) polymyxin E, (**E**) PAMP-12, (**F**) substance P, (**G**) BAM 8-22, (**H**) chloroquine, (**I**) compound 48/80, (**J**) poly-L-lysine, (**K**) atracurium, (**L**) tubocurarine, (**M**) TAN-67, or (**N**) leu-enkephalin. Arrows indicate injection of drug at 20 s, followed by injection of 50 µM ATP at 110 s as a positive control. Shaded regions indicate s.d. of technical replicates. EV plots are duplicated from Fig. S2.

# Figure S9

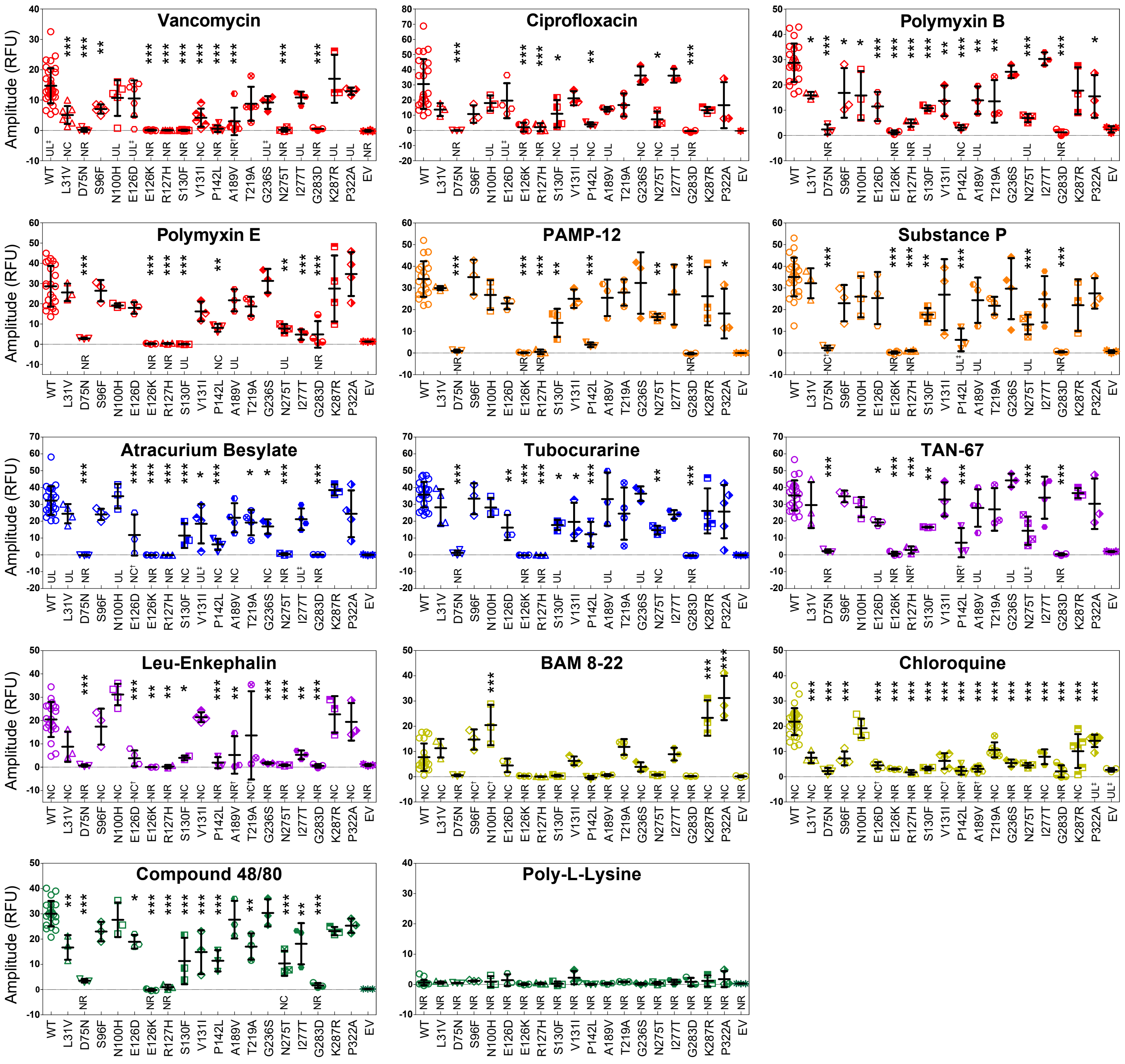


**Figure S9. Maximum amplitudes at the highest drug concentration.** The maximum responses to 1 mM vancomycin, 1 mM ciprofloxacin, 1 mM polymyxin B, 1 mM polymyxin E, 0.1 mM PAMP-12, 0.1 mM substance P, 1 mM atracurium besylate, 1 mM tubocurarine, 0.1 mM TAN-67, 1 mM leu-enkephalin, 0.1 mM BAM 8-22, 1 mM chloroquine, 3 μg/ml compound 48/80, and 30 μg/ml poly-L-lysine are shown. NR, NC, and UL labels are reproduced from Fig. 5. Mutants were compared to WT by one-way ANOVA and Dunnett's post-test: * $p < 0.05$; ** $p < 0.01$; *** $p < 0.001$. Each point represents an independent experiment, and error bars show overall mean ± SD.

# Figure S10

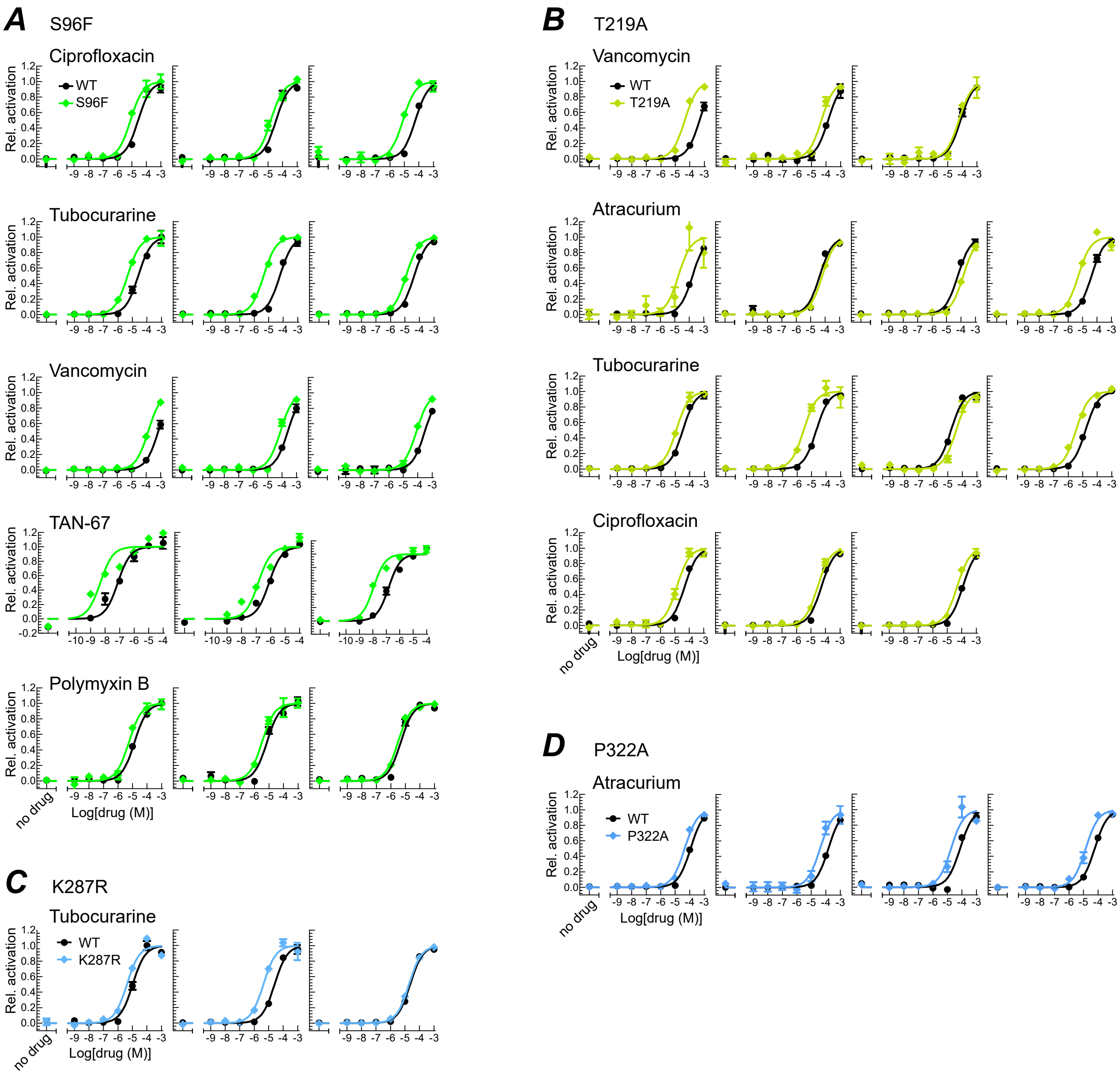


**Figure S10. Dose response curves for potential gain of function variants.** Replicate dose response curves are shown for variant–ligand combinations identified either by one-way ANOVA (Fig 5) or paired t-test (Fig 8), together with matched WT data from the same plate. Points represent mean ± SEM of three technical replicates.
